# Mental Health Surveillance Among Children — United States, 2013–2019

**DOI:** 10.15585/mmwr.su7102a1

**Published:** 2022-02-25

**Authors:** Rebecca H. Bitsko, Angelika H. Claussen, Jesse Lichstein, Lindsey I. Black, Sherry Everett Jones, Melissa L. Danielson, Jennifer M. Hoenig, Shane P. Davis Jack, Debra J. Brody, Shiromani Gyawali, Matthew J. Maenner, Margaret Warner, Kristin M. Holland, Ruth Perou, Alex E. Crosby, Stephen J. Blumberg, Shelli Avenevoli, Jennifer W. Kaminski, Reem M. Ghandour, Contributor, Leah N. Meyer

**Affiliations:** ^1^Division of Human Development and Disability, National Center on Birth Defects and Developmental Disabilities, CDC; ^2^Office of Epidemiology and Research, Maternal and Child Health Bureau, Health Resources and Services Administration, Rockville, Maryland; ^3^Division of Health Interview Statistics, National Center for Health Statistics, CDC; ^4^Division of Adolescent and School Health, National Center for HIV, Viral Hepatitis, STD, and TB Prevention, CDC; ^5^Division of Surveillance and Data Collection, Center for Behavioral Health Statistics and Quality, Substance Abuse and Mental Health Services Administration, Rockville, Maryland; ^6^Division of Violence Prevention, National Center for Injury Prevention and Control, CDC; ^7^Division of Health Nutrition Examination Surveys, National Center for Health Statistics, CDC; ^8^Division of Vital Statistics, National Center for Health Statistics, CDC; ^9^Division of Overdose Prevention, National Center for Injury Prevention and Control, CDC; ^10^Office of the Director, National Center on Birth Defects and Developmental Disabilities, CDC; ^11^Division of Injury Prevention, National Center for Injury Prevention and Control, CDC; ^12^National Institute of Mental Health, Bethesda, Maryland; Census Bureau.

## Abstract

Mental health encompasses a range of mental, emotional, social, and behavioral functioning and occurs along a continuum from good to poor. Previous research has documented that mental health among children and adolescents is associated with immediate and long-term physical health and chronic disease, health risk behaviors, social relationships, education, and employment. Public health surveillance of children’s mental health can be used to monitor trends in prevalence across populations, increase knowledge about demographic and geographic differences, and support decision-making about prevention and intervention. Numerous federal data systems collect data on various indicators of children’s mental health, particularly mental disorders. The 2013–2019 data from these data systems show that mental disorders begin in early childhood and affect children with a range of sociodemographic characteristics. During this period, the most prevalent disorders diagnosed among U.S. children and adolescents aged 3–17 years were attention-deficit/hyperactivity disorder and anxiety, each affecting approximately one in 11 (9.4%–9.8%) children. Among children and adolescents aged 12–17 years, one fifth (20.9%) had ever experienced a major depressive episode. Among high school students in 2019, 36.7% reported persistently feeling sad or hopeless in the past year, and 18.8% had seriously considered attempting suicide. Approximately seven in 100,000 persons aged 10–19 years died by suicide in 2018 and 2019. Among children and adolescents aged 3–17 years, 9.6%–10.1% had received mental health services, and 7.8% of all children and adolescents aged 3–17 years had taken medication for mental health problems during the past year, based on parent report. Approximately one in four children and adolescents aged 12–17 years reported having received mental health services during the past year. In federal data systems, data on positive indicators of mental health (e.g., resilience) are limited. Although no comprehensive surveillance system for children’s mental health exists and no single indicator can be used to define the mental health of children or to identify the overall number of children with mental disorders, these data confirm that mental disorders among children continue to be a substantial public health concern. These findings can be used by public health professionals, health care providers, state health officials, policymakers, and educators to understand the prevalence of specific mental disorders and other indicators of mental health and the challenges related to mental health surveillance.

## Introduction

Mental health is a broad label that encompasses a range of mental, emotional, social, and behavioral functioning. Mental health, like physical health, occurs along a continuum from good to poor and varies over time, in different conditions, and at different ages (*1*–*3*). Good mental health in children includes indicators such as the timely achievement of developmental milestones, healthy social and emotional development, and effective regulatory and coping skills; mentally healthy children function well in various settings including the home, school, and community (*4*–*7*). Poor mental health and patterns of symptoms that are severe, are persistent, and cause impairment or dysfunction can develop into mental disorders (*1*). Mental disorders are defined by the *Diagnostic and Statistical Manual of Mental Disorders, 5th Edition* (DSM-5) as clinically significant cognitive, emotional regulation, or behavior disturbances that reflect dysfunction in psychological, biological, or developmental mental function processes (*1*). Mental disorders are typically conceptualized as categorical (i.e., above or below a clinical cutoff of symptom or impairment scales), and children receive a diagnosis of a disorder when they have specific symptoms that meet specified criteria (*1*). Common mental disorders in children include anxiety, depression, attention-deficit/hyperactivity disorder (ADHD), and behavioral disorders (*8*). Good mental health is not simply the absence of a mental disorder; persons with diagnosed mental disorders can still have good mental health (e.g., if receiving adequate treatment and support) (*3*,*7*,*9*).

Throughout life, mental health and mental disorders are associated with immediate and long-term measures of physical health and chronic disease and with social determinants of health such as racial and ethnic minority status and any associated racial bias, social relationships, presence or absence of crime, and factors that determine access to resources such as education level, income level, and employment status (*10*–*22*). These and other social determinants of health that affect the environments in which children develop contribute to wide health disparities (*23*). Because of the direct connection between mental and physical health, promoting mental health, particularly in the context of social determinants of health, is essential to promoting health equity (*24*). Research has documented that policies and programs provided during childhood that improve children’s mental health also improve longer-term health and functioning and also might prevent children from developing a diagnosable disorder (*2*,*25*–*27*). Thus, promoting good mental health and addressing mental disorders among children are critical public health issues. Data on indicators of good and poor mental health, including mental disorders, can indicate where mental health promotion strategies are needed and how programs are affecting the mental health of the population.

Public health surveillance focuses on determining the prevalence of health conditions, can be used to monitor trends and changes in prevalence across subpopulations, and increases knowledge about sociodemographic and geographic differences in health indicators, which in turn increases knowledge of social determinants of health that affect health equity (*23*,*24*). Thus, public health surveillance provides the foundation for decision-making (*28*). Although mental health has increasingly become a focus of public health, surveillance efforts regarding mental health and mental disorders both among adults and children have faced various challenges, including insufficient timeliness; limited availability of data sources, particularly for state and local data; and lack of measures that are consistent and include a full set of specific disorders (*8*,*29*,*30*). For example, attempts at monitoring progress on effective treatments for mental disorders in the United States are often limited because of lack of adequate data sources. Treatment for ADHD or autism spectrum disorder (ASD) has been monitored using the National Survey of Children’s Health, and treatment for a major depressive episode (MDE) can be monitored with data from the National Survey of Drug Use and Health; however, treatment for anxiety, behavior problems, and trauma have not been monitored in national surveillance efforts (*31*). Challenges associated with surveillance of children’s mental health might include a separation among public health and mental health agencies at the federal, state, and local levels; stigma and privacy related to mental health data collection; and varying case definitions across surveillance systems (*8*,*29*).

Estimates from previous surveillance efforts and research studies indicate that approximately one in five children and adolescents experience a mental disorder each year (*32*,*33*); approximately two in five children and adolescents will meet criteria for a mental disorder by age 18 years (*34*,*35*), and one half of mental disorders have an onset before age 14 years (*32*). Although children in all sociodemographic groups are affected by mental disorders, the prevalence of different disorders varies by the child’s sex, age, residence (e.g., urban versus rural areas), race or ethnicity, and other sociodemographic characteristics (*8*,*33*,*34*,*36*,*37*). Prevalence estimates of diagnosed mental disorders have increased since 2000 for ADHD (*38*), anxiety (*17*), ASD (*38*), and depression (*19*). Similarly, since 2000, symptoms of mental disorders and indicators of poor mental health, including reports by youths of feeling sad or hopeless, suicidal ideation, and suicide attempts, as well as suicides among adolescents, have increased (*39*,*40*). During 2011–2019, suicide was the second leading cause of death among persons aged 10–29 years in the United States (*41*).

A 2013 report described federal surveillance efforts that included measures of children’s mental health and mental disorders (*8*). The report identified gaps in children’s mental health surveillance, including the need for 1) standard case definitions of mental disorders to improve comparability and reliability of estimates across surveillance systems; 2) surveillance of mental disorders among preschool-age children; and 3) surveillance of anxiety disorders (overall and by specific type), bipolar disorder, and other mental disorders that occur less commonly in children. Since then, available information about children’s mental health has increased. For example, although the need for standard case definitions has not yet been systematically addressed, more attention has been paid to the mental health of preschool-age children (*36*,*42*–*45*) and the prevalence of anxiety in children (*17*,*42*), and understanding of the impact of health equity on the development and diagnosis of mental disorders has increased (*2*,*37*,*46*,*47*).

In addition to increased attention to mental health, prevalence estimates might reflect revised diagnostic criteria published in DSM-5 in 2013 (*1*). Surveillance also might be affected by policy changes at the national and state levels regarding access to care, including the promotion of integrating primary and behavioral health services (*48*–*50*) and specific provisions for children with preexisting conditions (e.g., the Mental Health Parity and Addiction Equity Act and the Patient Protection and Affordable Care Act) (*51*–*54*).

This report updates and expands the 2013 surveillance report on mental health among children (*8*). Similar to the 2013 report, this report provides an overview of nine federal surveillance systems that collect data related to children’s mental health in the United States and the most recent estimates (2013–2019) available from these systems, including estimates according to selected sociodemographic characteristics linked to social determinants of health, such as age, sex, race and ethnicity, economic resources, parent education, access to health insurance, and geographic classification (*23*). In addition, whereas the 2013 report focused on national estimates of mental disorders and indicators of poor mental health among children, this report also includes data on 1) receipt of mental health services among children, 2) positive indicators of mental health, and 3) state-level estimates. First, this report includes data on receipt of mental health services among all children. These data are an indicator of the impact of mental disorders or symptoms of mental disorders on the service system, the costs associated with mental disorders, and access to specialized health services. Describing patterns of mental health services by subgroups might identify gaps in access to services or treatment for mental disorders and provide information to address health inequities (*30*,*55*,*56*). Second, this report includes several positive indicators of mental health that are measured on a continuum to provide a more inclusive picture of children’s mental health status. Third, this report includes state-level estimates when possible. State estimates are important because they describe the heterogeneity of smaller geographic units, can reflect the results of state-level practices and policies, and provide actionable information for program planning and resource allocation at a more local level (*57*,*58*). The findings in this report can be used by public health professionals, health care providers, state health officials, policymakers, and educators to understand the prevalence of specific mental disorders and other indicators of mental health and the challenges related to mental health surveillance.

## Methods

### Description of Surveillance Systems 

Nine data systems with indicators of children’s mental health were identified, including the Autism and Developmental Disabilities Monitoring Network (ADDM), the National Health and Nutrition Examination Survey (NHANES), the National Health Interview Survey (NHIS), the National Survey of Children’s Health (NSCH), the National Survey on Drug Use and Health (NSDUH), the National Violent Death Reporting System (NVDRS), the National Vital Statistics System (NVSS), the School-Associated Violent Death Surveillance System (SAVD-SS), and the national Youth Risk Behavior Survey (YRBS). Each of the data systems was designed to address different objectives, and the systems vary in methods (e.g., in-person interview, online questionnaire, vital statistics data, and parent report or self-report). Each system assesses different aspects of mental health, and the specific indicators included vary by survey; for example, although four systems include indicators of depression, the specific indicator is unique to each system. The data include persons ranging in age from 6 months to 19 years; although 17 years was the maximum age for most surveys, YRBS also included high school students aged ≥18 years, and data on suicides included persons aged 10–19 years for consistency with how these data are typically presented. Parents, guardians, and caregivers who answered survey questions as proxies for youths are collectively referred to as parents in this report. Following is a detailed description of the nine federal data systems and their associated data that are presented in this report, as well as a summary of each system (Tables 1 and 2). State-level ranges are presented when available; individual state prevalences are also available in the supplementary tables (Supplementary Tables 1–4; https://stacks.cdc.gov/view/cdc/113924). The most recent data available at the time the report was written are described and presented. 

**TABLE 1:** Federal surveys and surveillance systems that collect data on children’s mental health — United States

Name, website, sponsor	Description	Method of data collection	Topics related to children	Child-related mental health topics and questions	Populations and periodicity
**Autism and Developmental Disabilities Monitoring (ADDM) Network** https://www.cdc.gov/ncbddd/autism/addm.html CDC, National Center on Birth Defects and Developmental Disabilities	– The ADDM Network is a group of programs funded by CDC to determine the prevalence of ASDs in U.S. communities. – The ADDM sites collect data using the same surveillance methods, which are modeled after CDC’s Metropolitan Atlanta Developmental Disabilities Surveillance Program.	– Screening and abstraction of existing health and education records containing professional assessments of the child's developmental progress at health care or education facilities	– Baseline data on ASD prevalence in participating communities; comparisons between different groups of children and different areas of the country – Characteristics of the population of children with ASDs – Changes in ASD identification over time – Impact of autism and related conditions in selected U.S. communities – Risk factors	– Describes the population of children with ASDs in terms of 1) documented co-occurring conditions and 2) timing of earliest evaluation and diagnosis	– Ongoing since 2000, with data available for even-numbered years through 2016 – Population-based, geographically defined communities in different U.S. states, selected through competitive process – In 2016, monitored ASD among 275,000 children aged 8 yrs and 72,000 aged 4 yrs
**National Health and Nutrition Examination Survey (NHANES)** https://www.cdc.gov/nchs/nhanes CDC, National Center for Health Statistics	– NHANES is an ongoing cross-sectional survey designed to assess the health and nutritional status of noninstitutionalized persons of all ages in the United States.	– In-person household interviews by trained interviewers using computer-assisted personal interviewing; private interviews in medical examination center – Nutritional assessments – Laboratory tests – Physical examinations	– Nutrition and nutritional disorders – Environmental risk factors – Health care use – Mental, behavioral, and emotional problems of children – Infectious diseases – Weight and physical activity – Dietary supplements and prescription medications – Medical conditions and health indicators, including disability (measured and self-report) – Health insurance status and type	– Alcohol and drug use – Depression (PHQ-9 since 2005) – Use of mental health care services – Psychotropic medication use – Functional limitations caused by long-term physical, mental, and emotional conditions or illness – Mental disorder diagnosis for specific disorders using the Diagnostic Interview Schedule for Children (1999–2004) – Mentally unhealthy days (2001–2014)	– Ongoing since 1999 – Nationally representative sample – 5,000 persons per year, including approximately 1,600 persons aged 3–17 yrs – Oversampling, which changes periodically; in 2013–2014, 2015–2016, and 2017–2018, oversampled persons with low incomes and Hispanic, Black, and Asian persons
**National Health Interview Survey (NHIS)** https://www.cdc.gov/nchs/nhis CDC, National Center for Health Statistics	– NHIS monitors a broad range of health topics among the U.S. civilian noninstitutionalized population, including children aged 0–17 yrs.	– In-person household interview (of parent or knowledgeable adult who lives in the household and answers on behalf of one randomly selected child in the family) conducted by trained interviewers using computer-assisted personal interviewing, with telephone follow-up when needed	– Health status, selected health conditions and illnesses, disability, selected educational services, health insurance status and type, and health care access	– ASD (ever and current) and ADHD or ADD (ever and current) – Use of mental health care or counseling – Strengths and Difficulties Questionnaire	– Ongoing household survey representative of the U.S. civilian noninstitutionalized population – Has collected data for children annually since 1997
**National Survey of Children’s Health (NSCH)** https://mchb.hrsa.gov/data-research/national-survey-childrens-health Health Resources and Services Administration, Maternal and Child Health Bureau	– NSCH is a cross-sectional, address-based survey conducted by the Census Bureau that collects information on the health and well-being of children aged 0–17 yrs and related health care, family, and community-level factors that can influence health. – Data from NSCH are the only source of both national- and state-level estimates on specified measures of child health. – NSCH was redesigned for 2016, combining two previously separate Maternal and Child Health Bureau quadrennial surveys, the National Survey of Children with Special Health Care Needs, and NSCH.	– Parent/caregiver-administered questionnaire online and on paper	– Physical and mental health status – Health and functional status, including approximately 20 current or lifelong conditions – Health insurance status, type, and adequacy – Access to and use of health care services – Preventive and specialty care – Medical home – School readiness (3–5 yrs) – Transition to adult health care (12–17 yrs) – Family health and activities – Impact of child’s health on family – Parent/caregiver health status – Parent/caregiver perceptions of neighborhood characteristics – Access to community-based services	– Diagnosis (ever and current) and severity of depression, anxiety problems, behavioral or conduct problems, Tourette syndrome, and ADD or ADHD – Diagnosis (ever and current) and severity of ASD, as well as age at diagnosis and type of provider who made first diagnosis – Current medication treatment for autism, ASD, Asperger’s disorder, PDD, and ADHD – Receipt of behavioral treatment in past year for ASD and ADHD – Receipt of treatment or counseling in past year from a mental health professional – Medication use in past year because of difficulties with emotions, concentration, or behavior – Positive indicators (indicate child is flourishing): affectionate and tender, bounces back quickly, smiles and laughs, interest in and curiosity about learning new things, works to finish tasks, stays calm and in control when faced with a challenge	– Annual survey representative of noninstitutionalized children aged 0–17 yrs at the national and state levels
**National Survey on Drug Use and Health (NSDUH)** https://www.samhsa.gov/data/data-we-collect/nsduh-national-survey-drug-use-and-health SAMHSA, Center for Behavioral Health Statistics and Quality	– NSDUH provides up-to-date information on tobacco, alcohol, and drug use; mental health; and other health-related issues in the United States. – Information from NSDUH is used to support prevention and treatment programs, monitor substance use trends, estimate the need for treatment, and guide public health policy.	– In-person household interviews by trained interviewers using audio computer-assisted self-interviewing (ACASI)	– Timing and frequency of use of tobacco products, alcohol, marijuana, cocaine, heroin, inhalants, hallucinogens, and prescription drugs – Risk and protective factors – Health care use – Health insurance status and type	– Major depressive episode ever (lifetime) and in past year – Substance use disorder, overall and by substance type (e.g., illicit drug use disorder or alcohol use disorder) and treatment – Level of impairment resulting from major depressive episodes – Treatment for depression – Mental health service use – Parental mental illness, substance use, and substance use disorder	– Began in 1971 and has been administered by SAMHSA since 1992 – Conducted every year in all 50 states and the District of Columbia, with approximately 70,000 people aged ≥12 yrs interviewed each year – National and state representative sample
**National Violent Death Reporting System (NVDRS)** https://www.cdc.gov/violenceprevention/datasources/nvdrs/index.html CDC, National Center for Injury Prevention and Control	– NVDRS is a state-based active surveillance system that collects data on the characteristics and circumstances associated with violence-related deaths in participating states and territories.	– Death certificates, coroner/medical examiner reports, law enforcement reports, and secondary sources (e.g., child fatality review team data, supplemental homicide reports, hospital data, and crime laboratory data)	– Violent deaths: suicides, homicides, deaths from legal intervention (a subtype of homicide), child maltreatment deaths, and intimate partner homicides – Other deaths: deaths from undetermined intent and unintentional firearm deaths	– Numerous precipitating circumstances that are associated with suicide, including history of mental health problems or substance abuse; current diagnosis or treatment for mental health problems; history of suicide, thoughts, plans, or attempts; interpersonal problems; life stressors; and suicide disclosure	– Expanded in 2018 with the addition of 10 new states (Arkansas, Florida, Idaho, Mississippi, Montana, North Dakota, South Dakota, Tennessee, Texas, and Wyoming) that began data collection in 2019; in 2019, all 50 states, the District of Columbia, and Puerto Rico collected data for the system
**National Vital Statistics System (NVSS)** https://www.cdc.gov/nchs/nvss/deaths.htm CDC, National Center for Health Statistics	– Vital statistics mortality data are a fundamental source of demographic, geographic, and cause-of-death information in the United States. The data are used to present characteristics of those dying in the United States, to understand causes of death, to determine life expectancy, and to compare mortality trends with those in other countries.	– Death certificates, which are completed by funeral directors, attending physicians, medical examiners, and coroners, with causes of death processed in accordance with the *International Classification of Diseases, Tenth Revision*	– Underlying cause of death – Multiple causes of death – Year, month, and day of week of death – Residence of decedent (state, county, city, population size) – State and county of occurrence – Demographic information about decedent (e.g. age at death, sex, education, race, ethnicity, marital status, and state of birth)	– Information for children who die as a result of suicide or from other causes of death that are related to mental health (e.g., drug overdose)	– Ongoing since 1900 to present – Did not include all states before 1933 – Includes information on all deaths occurring in the United States – Final annual data published yearly; provisional data released quarterly, monthly, and weekly for specific cause of death
**School-Associated Violent Death Surveillance System (SAVD-SS)** https://www.cdc.gov/violenceprevention/youthviolence/schoolviolence/savd.html CDC, National Center for Injury Prevention and Control	– SAVD-SS is an active surveillance system that collects data on the characteristics and circumstances surrounding school-associated violent deaths (homicides, suicides, and legal intervention deaths) that occur in and around school settings throughout the United States.	– Potential SAVD cases are identified through a systematic media scan of newspaper and broadcast media databases via LexisNexis using keywords to capture reports of violent deaths. Cases are then confirmed with local law enforcement or school officials familiar with the incident to ascertain whether they meet SAVD-SS inclusion criteria, and the law enforcement report is obtained when possible. – Demographic and circumstance data are abstracted from law enforcement reports, death certificates, coroner and medical examiner reports (if contained in the law enforcement report), interviews with law enforcement and school officials, or articles published in the media when a reliable source is cited (i.e., a law enforcement or school official or judicial proceedings regarding the incident).	– Circumstances that precipitated incidents (e.g., dating partner problems, other relationship problems, disciplinary issues, bullying or other problems experienced in the school setting, and history of criminal or legal problems) – Potential risk factors for perpetration and victimization – Possible prevention measures – Warning signs	– Whether the victim and perpetrator had a suspected or diagnosed mental health condition, suicidal ideation, or history of suicide attempt – Whether victim and perpetrator were using alcohol or substances at the time of death or regularly used alcohol or substances – Whether victim and perpetrator were victimized or had perpetrated violence in the past	– Since 1994 – Identifies and collect data on all U.S. incidents in which lethal violence occurs 1) on the campus of a functioning public or private primary or secondary school, 2) while the victim was on the way to or from regular sessions at such a school, or 3) while the victim was attending or traveling to or from an official school-sponsored event – Beginning in 2021, will collect data via NVDRS (described in previous row)
**National Youth Risk Behavior Survey (YRBS)** https://www.cdc.gov/yrbss CDC, National Center for HIV, Viral Hepatitis, STD, and TB Prevention	– The Youth Risk Behavior Surveillance System monitors health behaviors and experiences among U.S. high school students that contribute to the leading causes of mortality, morbidity, and social problems among youths and adults. – The system includes a national YRBS conducted by CDC and separate state, tribal, territorial, and local school district YRBSs.	– Anonymous, school-based survey	– Behaviors that contribute to unintentional injuries and violence – Tobacco use – Alcohol and other drug use – Sexual behaviors that contribute to unintended pregnancy and sexually transmitted infections – Unhealthy dietary behaviors – Inadequate physical activity – Prevalence of obesity and asthma and other health-related behaviors and experiences	– Persistent feelings of sadness or hopelessness – Suicidal ideation and suicide attempts	– Biennial since 1991 (odd years) – Nationally representative samples of public and private high school students (grades 9–12) – State, tribal, territorial, and local school district data also available

**Abbreviations:** ADD = attention-deficit disorder; ADHD = attention-deficit/hyperactivity disorder; ASD = autism spectrum disorder; PDD = pervasive developmental disorder; PHQ-9 = nine-item Patient Health Questionnaire; SAMHSA = Substance Abuse and Mental Health Services Administration.

**TABLE 2:** Mental disorders and indicators among persons aged 0–19 years, by surveillance system and age group — United States, 2013–2019

Disorder or indicator	Surveillance system and age group (yrs)								
	ADDM Network	NHANES	NHIS	NSCH	NSDUH	NVDRS	NVSS	SAVD-SS	National YRBS
	8	12‒17	3‒17	0‒17	12‒17	10‒19	10‒19	10–18	~14‒18*
Attention-deficit/ hyperactivity disorder	NA	NA	Parent report of diagnosis by a health care provider (ever, current)	Parent report of diagnosis by a health care provider (ever, current)	NA	NA	NA	NA	NA
Behavior problems	NA	NA	NA	Parent report of diagnosis by a health care provider (ever, current)	NA	NA	NA	Law enforcement or school official report of decedents’ history of behavior problems at home or in school	NA
Depression	NA	Youth self-report of current depression (depression during past 2 wks, score of ≥10 on PHQ-9 depression screener)	NA	Parent report of diagnosis by a health care provider (ever, current)	Youth self-report of major depressive episode (ever, past year)^†^	NA	Depression might be inconsistently reported on the death certificate	Law enforcement or school official report of decedents’ history of depressed mood or documented diagnosis of depression	Youth self-report of feeling so sad or hopeless almost every day for ≥2 wks in a row that they stopped doing some usual activities
Anxiety	NA	NA	NA	Parent report of diagnosis by a health care provider (ever, current)	NA	NA	Anxiety might be inconsistently reported on the death certificate	NA	NA
Autism spectrum disorder	Medical record abstraction, surveillance case criteria met^§^	NA	Parent report of diagnosis by a health care provider (ever, current)	Parent report of diagnosis by a health care provider (ever, current)	NA	NA	NA	NA	NA
Tourette syndrome	NA	NA	NA	Parent report of diagnosis by a health care provider (ever, current)	NA	NA	NA	NA	NA
Substance use disorder	NA	NA	NA	—^¶^	Youth self-report of dependence on or abuse of alcohol or one or more illicit drugs (in past year) based on DSM-IV criteria	NA	Substance use disorder might be inconsistently reported on the death certificate	NA	NA
Suicidality	NA	NA	NA	NA	NA	Death records; deaths per 100,000	Death records; deaths per 100,000	Surveillance of school-associated suicide based on systematic media scan of computerized newspaper and broadcast media databases and confirmation with local law enforcement	Youth self-report of seriously considering attempting suicide, making a suicide plan, attempting suicide ≥1 time, and making a suicide attempt ≥1 time that resulted in injury, poisoning, or overdose that had to be treated by a physician or nurse (during the 12 months before the survey for all four measures)
								Law enforcement or school official report of decedents’ history of suicidal thoughts and suicide attempts	
Treatment	NA	Parent report of child currently using psychotherapeutic agents (in past 30 days)	Parent report of child receiving mental health consultation with professional** or general physician^††^	Parent report of child receiving mental health treatment by a professional^§§^ and past year medication for mental health problems^¶¶^	Youth self-report of child receiving mental health services (specialty or nonspecialty)	NA	NA	Law enforcement or school official report of decedents’ history of mental health treatment provided by a counselor (including a school counselor) or clinician	NA
		Parent report of child receiving mental health consultation with a professional**							
Positive indicators	NA	NA	NA	Parent report of affection, resilience, and positivity (for children aged 6 mos‒5 yrs); curiosity (6 mos–17 yrs); and persistence and self-control (6–17 yrs)	NA	NA	NA	NA	NA

**Abbreviations:** ADDM = Autism and Developmental Disabilities Monitoring; DSM-IV = *Diagnostic and Statistical Manual of Mental Disorders, 4th Edition*; MDE = major depressive episode; NA = not applicable; NHANES = National Health and Nutrition Examination Survey; NHIS = National Health Interview Survey; NSCH = National Survey of Children’s Health; NSDUH = National Survey on Drug Use and Health; NVDRS = National Violent Death Reporting System; NVSS = National Vital Statistics System; PHQ-9 = nine-item Patient Health Questionnaire; SAVD-SS = School-Associated Violent Death Surveillance System; YRBS = Youth Risk Behavior Survey.

* Survey participants were public and private high school students in grades 9–12 (i.e., primarily aged 14–18 years).

^†^ Ever MDE: reported at least five or more of nine symptoms nearly every day in the same 2-week period during their lifetime, in which at least one of the symptoms was depressed mood or loss of interest or pleasure in daily activities; MDE in past year: 1) had ever had an MDE, as well as 2) had a period of time in the past 12 months when they felt depressed or lost interest or pleasure in daily activities for ≥2 weeks and 3) during this period of ≥2 weeks, they had some of the other problems they reported associated with ever having had an MDE.

^§^ Case definition based on *Diagnostic and Statistical Manual of Mental Disorders, 5th Edition* criteria for autism spectrum disorder. Clinicians applied the case definition through a review of information systematically collected from developmental evaluations completed by medical and educational service providers in the community.

^¶^ NSCH included a question about parent report of a health care provider diagnosis for substance use disorder for children aged 6‒17 years in the 2016–2019 surveys. These data are not included in this report because NSCH removed this item as of 2020 due to small samples and concerns about validity with parental report.

** “During the past 12 months, have you seen or talked to...a mental health professional such as a psychiatrist, psychologist, psychiatric nurse, or clinical social worker...about child's health?”

^††^ “Did you see or talk to this general doctor because of an emotional or behavioral problem that [child] may have?”

^§§^ “During the past 12 months, has this child received any treatment or counseling from a mental health professional?”

^¶¶^ “During the past 12 months, has this child taken any medication because of difficulties with his or her emotions, concentration, or behavior?”

#### Autism and Developmental Disabilities Monitoring Network

The ADDM Network is an active surveillance program conducted by CDC that provides estimates of the prevalence of ASD among children aged 4–8 years whose parents or guardians live in geographically defined areas of the United States. The ADDM Network has reported population-based estimates of ASD prevalence among children aged 8 years in even-numbered years since 2000. The most recent data at the time the report was written are from 11 geographically diverse sites that conducted population-based ASD surveillance for 2016. Surveillance is conducted in two phases. The first phase involves review and abstraction of comprehensive evaluations that were completed by medical and educational service providers in the community. In the second phase, experienced clinicians systematically review all abstracted information to determine ASD case status. The case definition is based on ASD criteria described in DSM-5 (*1*).

Although not nationally representative, ASD prevalence estimates are available by site, and the sample size allows for estimation and comparisons of sociodemographic characteristics within each participating community. Although individual-level ADDM Network data are not publicly available, various site-level or group-level results are available (https://www.cdc.gov/ncbddd/autism/addm.html), and CDC has an interactive website that allows ADDM data to be visualized or downloaded along with other state-based sources of ASD prevalence data (https://www.cdc.gov/ncbddd/autism/data/index.html).

The results presented in this report are based on 2016 data. Among 275,409 children aged 8 years living in the geographically defined areas comprising the 11 sites, a total of 5,108 children were identified as having ASD. For other mental health indicators, the ADDM Network sites review children’s medical and educational evaluations and collect information on co-occurring diagnoses of mental disorders such as ADHD, anxiety, and depression among children with ASD.

#### National Health and Nutrition Examination Survey

NHANES is a continuous cross-sectional survey on health and nutrition conducted by CDC (https://www.cdc.gov/nchs/nhanes). NHANES uses a multistage probability household sampling design to obtain nationally representative estimates of health indicators for the U.S. civilian noninstitutionalized population of all ages. Since 2011, NHANES has oversampled Hispanic, non-Hispanic Asian (Asian), and non-Hispanic Black or African American (Black) persons to increase the reliability and precision of estimates for these subgroups. Data are collected through examination and assessments in a mobile examination center (MEC), as well as interviews in the home. Health indicators have included cardiovascular disease, diabetes, obesity, environmental exposures, infectious diseases and vaccination, mental health, oral health, dietary intake, and supplement and prescription medication use (*59*). NHANES data are released in 2-year cycles. Most data are available online (https://www.cdc.gov/nchs/nhanes); restricted data, such as depression symptoms for children and adolescents aged 12–17 years (collectively referred to as adolescents in this report), are available to researchers through the CDC Research Data Center (https://www.cdc.gov/rdc). For this report, the data have been pooled for NHANES cycles 2013–2014, 2015–2016, and 2017–2018. The 2019–2020 NHANES cycle was not completed because of the onset of the COVID-19 pandemic, and the data for the cycle collected in 2019 through March 2020 have been combined with the prepandemic 2017–2018 data for future analysis; however, these data were not available for this report. Across these cycles, overall NHANES interview response rates ranged from 71.0% to 51.9%; MEC examination response rates ranged from 68.5% to 48.8%. Response rates for adolescents and young adults aged 12–19 years were approximately 5 percentage points higher than the overall response rates in every cycle.

NHANES estimates for this report are based on data collected during the in-home interview and private in-person MEC interview. Since the 2005–2006 administration, NHANES has included the nine-item Patient Health Questionnaire (PHQ-9) to measure depression symptoms (*60*). The PHQ-9 screening instrument consists of nine questions about depression symptoms during the past 2 weeks followed by a single question that assesses associated impairment; the resulting scores are used to determine depression severity and range from 0 to 27 (*60*). Adolescent self-reports on depression symptoms were collected during the MEC interview. The PHQ-9 has been estimated to have a sensitivity of 89.5% and a specificity of 77.5% for detecting adolescents who meet the DSM-IV (*61*) criteria for major depression on the Diagnostic Interview Schedule for Children, Version IV (DISC-IV) (*62*,*63*). In this report, weighted estimates of depression are based on a score of ≥10 for adolescents aged 12–17 years who completed the MEC interview for NHANES during 2013–2018. In past NHANES cycles, other mental health assessments were administered to youths, such as diagnostic modules from DISC during 1999–2004 (*64*).

Information on use of mental health services, including receiving care from a mental health professional (ages 4–17 years) and use of psychotropic medication (ages 3–17 years), was obtained during the NHANES home interview, with self-reports from adolescents aged ≥16 years and from parents for those aged <16 years. Use of mental health services was assessed by the questions, “During the past 12 months, have you seen or talked to a mental health professional such as a psychologist, psychiatrist, psychiatric nurse, or clinical social worker about (his/her/your) health?” and “In the past 30 days, have you used or taken any medication for which a prescription is needed?” Those who responded “yes” to the medication question were asked to provide containers of all prescription medications, and interviewers recorded the product name. Products were identified using a proprietary, comprehensive database of prescription and certain nonprescription drugs (Lexicon Plus, Cerner Multum, Inc.) and classified according to a three-level classification scheme (*65*). Reported use of any of the following psychotropic medications resulted in the classification of the youth as using psychotropic medication: antidepressant medications; central nervous system stimulants; anxiolytics, sedatives, and hypnotics; antimanic medications; and antipsychotic medications. 

#### National Health Interview Survey

NHIS is a survey of a nationally representative sample of the civilian noninstitutionalized U.S. population and is conducted continuously throughout the year by CDC to monitor the health of the U.S. population (https://www.cdc.gov/nchs/nhis). NHIS is an in-person interview conducted in the respondent’s home; in some instances, a follow-up to complete the interview is conducted via telephone. For interviews completed during 1997–2018, families were identified within each sampled household, and a family member completed a brief questionnaire on selected demographic and broad health measures. From each family in the household, one adult and one child were randomly selected to receive a more detailed health questionnaire. A parent answered questions for one randomly selected child or adolescent aged 0–17 years. NHIS data are publicly available online (https://www.cdc.gov/nchs/nhis); restricted data are available to researchers through the NCHS Research Data Center (https://www.cdc.gov/rdc).

Data included in this report are pooled from the 2017 and 2018 surveys, which included 8,845 and 8,269 children and adolescents aged 0–17 years, respectively. The total household response rate was 66.5% in 2017 and 64.2% in 2018, and the final response rate for the sample child component was 60.6% in 2017 and 59.2% in 2018. 

NHIS has included questions on ASD and ADHD annually since 1997, with some changes in the ASD measure over time. For ADHD, parents were asked, “Has a doctor or health professional ever told you that [child] had attention-deficit/hyperactivity disorder (ADHD) or attention-deficit disorder (ADD)?” Since 2014, the ASD question wording has been consistently phrased as, “Has a doctor or health professional ever told you that [child] had autism, Asperger’s disorder, pervasive developmental disorder, or autism spectrum disorder?” Beginning in 2016, follow-up questions were added to determine current diagnoses: “Does [child] currently have attention-deficit/hyperactivity disorder (ADHD) or attention-deficit disorder (ADD)?” and “Does [child] currently have autism, Asperger’s disorder, pervasive developmental disorder, or autism spectrum disorder?”

To assess use of mental health services, NHIS included the following question on mental health care consultations for emotional or behavioral problems: “During the past 12 months, have you seen or talked to any of the following health care providers about [child’s] health? A mental health professional such as a psychiatrist, psychologist, psychiatric nurse, or clinical social worker?” In addition, for those who reported having consulted with a general physician in the past 12 months, a follow-up question was used to address whether the physician was consulted specifically for emotional or behavioral problems: “Did you see or talk to this general doctor because of an emotional or behavioral problem that [child] may have?” The denominator included all children, regardless of whether they had consulted a general physician in the past 12 months.

#### National Survey of Children’s Health

NSCH is an annual, cross-sectional, address-based survey that collects information on the health and well-being of children and adolescents aged 0–17 years, as well as related health care, family, and community factors that can influence health (https://mchb.hrsa.gov/data-research/national-survey-childrens-health). NSCH is funded and directed by the Health Resources and Services Administration’s Maternal and Child Health Bureau (HRSA MCHB), with co-sponsorship from CDC, the U.S. Department of Agriculture, and others, and is conducted by the Census Bureau using both online and paper methods. Data are collected from a parent in the household who is knowledgeable about the health and health care of one randomly selected child. Data provide both national and state estimates on key measures of child health. The data are subject to error arising from various sources, including sampling and nonsampling errors. Additional general information is available (https://mchb.hrsa.gov/data/national-surveys), and NSCH data and questionnaires are publicly available online (https://mchb.hrsa.gov/data/national-surveys/questionnaires-datasets-supporting-documents).

NSCH was redesigned for 2016, combining two previously separate HRSA MCHB quadrennial surveys, the National Survey of Children with Special Health Care Needs (NS-CSHCN) and NSCH (*66*). Because of changes in the mode of data collection and sampling frame, as well as adjustments to item wording as needed, estimates from the redesigned 2016 survey cannot be directly compared with those from earlier years, nor can trend analyses using data collected before and after 2016 be conducted (*66*). Since 2016, NSCH data have been collected annually. A total of 50,212 questionnaires were completed for the 2016 NSCH, followed by 21,599 in 2017, 30,530 in 2018, and 29,433 in 2019. The overall weighted response rate was 40.7% in 2016, 37.4% in 2017, 43.1% in 2018, and 42.4% in 2019; the weighted interview completion rate (or the probability that a household that initiates the survey completes it) was 69.7%, 70.9%, 78.0%, and 79.5%, respectively. Approximately 75% of questionnaires were completed online each year.

NSCH assesses and reports data on the presence of diagnosed mental health problems or conditions in children and adolescents aged 0–17 years. Parents were asked about 1) depression, 2) anxiety problems, 3) behavioral or conduct problems, 4) autism or ASD, 5) Tourette syndrome, and 6) attention-deficit disorder (ADD) or ADHD. For each condition, parents were asked whether they had ever been told by a doctor or other health care provider that their child had the condition and whether the child currently had the condition; for behavioral or conduct problems, parents also were asked to consider reports from educators, including teachers and school nurses. For children with current problems or conditions, parents were asked to rate the severity of their child’s condition as mild, moderate, or severe. NSCH also assesses and reports data on receipt of mental health treatment among children and adolescents aged 0–17 years. Parents were asked whether their children received mental health treatment or counseling or took medications for a problem with emotions, concentration, or behavior. Additional treatment indicators that were available from NSCH only for a selected subset of disorders such as medication or behavioral treatment specifically for ADHD and ASD were not included. Data included in this report for diagnoses and treatment are from the 2016–2019 surveys.

NSCH also includes parent responses to items that can be used as positive indicators of mental health (*67*,*68*). For children aged 6 months–5 years, parents were asked how often the child 1) is affectionate and tender (labeled affection), 2) bounces back quickly when things do not go his or her way (labeled resilience), and 3) smiles and laughs a lot (labeled positivity). For children and adolescents aged 6 months–17 years, parents were asked how often the child shows interest and curiosity in learning new things (labeled curiosity). For children and adolescents aged 6–17 years, parents were asked how often the child 1) works to finish tasks that have been started (labeled persistence) and 2) stays calm and in control when faced with a challenge (labeled self-control). Beginning in 2018, response options for the positive indicators were always, usually, sometimes, and never. Children were considered to meet the indicator criteria if the parent answered usually or always. Data included in this report for positive indicators are from the 2018–2019 surveys.

#### National Survey on Drug Use and Health

NSDUH is the primary source of statistical information on the use of illicit drugs, alcohol and tobacco use, substance use disorders, MDEs, and receipt of mental health and substance use services among the civilian, noninstitutionalized population aged ≥12 years in the United States, all 50 states, and the District of Columbia (DC) (https://www.samhsa.gov/data/data-we-collect/nsduh-national-survey-drug-use-and-health). Conducted by the federal government since 1971 (annually since 1990), NSDUH is sponsored by the Substance Abuse and Mental Health Services Administration (SAMHSA). For the years analyzed, NSDUH collected data through in-person interviews of household residents, persons living in noninstitutional group settings, and civilians living on military bases. NSDUH collects data using audio computer-assisted self-interviewing (ACASI) for sensitive items in which respondents read or listen to questions through headphones and then enter their answers directly into an NSDUH laptop computer.

NSDUH is a state and nationally representative survey with approximately 150,000 addresses screened and approximately 67,500 respondents interviewed each year. Adolescents and young adults are oversampled. Additional general information about NSDUH is available (https://www.samhsa.gov/data/data-we-collect/nsduh-national-survey-drug-use-and-health), and NSDUH data are publicly available online (https://www.datafiles.samhsa.gov). In 2018, screening was completed at 141,879 addresses, and 67,791 completed interviews were obtained, including 16,852 interviews from adolescents aged 12–17 years. The weighted response rates were 73.3% for household screening, 66.6% for interviewing, and 48.8% overall (*69*). In 2019, screening was completed at 148,023 addresses, and 67,625 completed interviews were obtained, including 16,894 interviews from adolescents aged 12–17 years. The weighted response rates were 70.5% for household screening, 64.9% for interviewing, and 45.8% overall (*70*).

NSDUH uses ACASI to assess whether adolescents aged 12–17 years ever experienced an MDE (i.e., lifetime MDE) as defined by DSM-IV (*61*) or experienced an MDE in the past year. Lifetime MDE is defined as ever having at least five or more of nine symptoms of depression in the same 2-week period, in which at least one of the symptoms was a depressed mood or loss of interest or pleasure in daily activities. An adolescent was defined as having an MDE in the past year if they met all of the following criteria: 1) had ever had an MDE, as well as 2) had a period of time in the past 12 months when they felt depressed or lost interest or pleasure in daily activities for ≥2 weeks and 3) during this period of ≥2 weeks, they had some of the other problems they reported associated with ever having had an MDE.

NSDUH collects data on substance use disorders and types of substance use disorders (e.g., alcohol use disorder and illicit drug use disorder) annually. In the 2018 and 2019 surveys, substance use disorder was defined as meeting the DSM-IV criteria for either dependence or abuse for alcohol or one or more illicit drug. NSDUH also used DSM-IV dependence or abuse criteria to define alcohol use disorder and illicit drug use disorder (*61*). Additional information on the criteria NSDUH uses for substance use disorders are available online (*70*). Illicit drug use disorder included dependence or abuse of one or more of the following illicit drugs: marijuana, cocaine, heroin, hallucinogens, inhalants, methamphetamine, or prescription psychotherapeutic drugs that were misused (i.e., pain relievers, tranquilizers, stimulants, and sedatives). One clinical validation study compared the NSDUH instrument with the Pittsburgh Adolescent Alcohol Research Center’s Structured Clinical Interview (*71*). The level of agreement was fair to moderate overall, with sensitivity values of 81%–95% and specificity values of 48%–63% for substance abuse or dependence (*71*). The 2006 NSDUH Reliability Study, which compared responses on interviews conducted 5–15 days apart, reported a Kappa value of 0.67 for illicit drug or alcohol use disorders and was 0.62 for illicit drug use disorders (*72*).

The 2018 and 2019 NSDUH survey years included questions to estimate the use of mental health services among adolescents aged 12–17 years. In addition to asking about treatment for depression, the surveys also included questions about receipt of any services for emotional or behavioral problems not caused by substance use. The NSDUH interview section on use of mental health services among youths asks adolescent respondents whether they received any treatment or counseling in the 12 months before the interview in specialty and nonspecialty settings. Specialty mental health settings include services in outpatient or inpatient settings. Outpatient services include 1) a private therapist, psychologist, psychiatrist, social worker, or counselor; 2) a mental health clinic or center; 3) a partial day hospital or day treatment program; or 4) an in-home therapist, counselor, or family preservation worker. Inpatient or residential specialty mental health services in which adolescents stayed overnight or longer include services in a hospital or a residential treatment center. Nonspecialty mental health settings for adolescents include the education, general medical, juvenile justice, and child welfare settings. 

#### National Violent Death Reporting System

NVDRS is a population-based active surveillance system conducted by CDC to collect data on violent deaths that occur within participating states and territories (https://www.cdc.gov/violenceprevention/datasources/nvdrs/index.html). For the purposes of NVDRS, violence is defined as the intentional use of threatened or actual physical force or power against a person, group, or community that causes or is likely to cause injury, death, psychological harm, developmental harm (e.g., arrested physical, mental, intellectual, emotional, or social development), or deprivation. Violent deaths include child maltreatment deaths, intimate partner homicides and other homicides, suicides, and legal intervention deaths (i.e., when a decedent is killed by a police officer or other person with specified legal authority to use deadly force) (*73*). Unintentional firearm injury deaths and deaths of undetermined intent also are included in the system. States are required to begin entering all deaths into the online system within 4 months from the date the violent death occurred. States then have an additional 14 months from the end of the calendar year in which the violent death occurred to complete each incident record. Additional information on NVDRS is available (https://www.cdc.gov/violenceprevention/datasources/nvdrs/index.html).

NVDRS data collection began in 2003 with six participating states (Maryland, Massachusetts, New Jersey, Oregon, South Carolina, and Virginia). Seven additional states began data collection in 2004 (Alaska, Colorado, Georgia, North Carolina, Oklahoma, Rhode Island, and Wisconsin), three in 2005 (Kentucky, New Mexico, and Utah), two in 2010 (Ohio and Michigan), and 14 in 2015 (Arizona, Connecticut, Hawaii, Illinois, Indiana, Iowa, Kansas, Maine, Minnesota, New Hampshire, New York, Pennsylvania, Vermont, and Washington). Eight additional states (Alabama, California, Delaware, Louisiana, Missouri, Nebraska, Nevada, and West Virginia), DC, and Puerto Rico began data collection in 2017. California began collecting data in 2005 but ended data collection in 2009; in 2017, California collected NVDRS data from all three required sources (i.e., death certificates, coroner or medical examiner reports, and law enforcement reports) from four counties (Los Angeles, Sacramento, Shasta, and Siskiyou). NVDRS received funding in 2018 for a nationwide expansion that included the remaining 10 states (Arkansas, Florida, Idaho, Mississippi, Montana, North Dakota, South Dakota, Tennessee, Texas, and Wyoming), which began data collection in 2019. CDC now provides NVDRS funding to all 50 states, DC, and Puerto Rico (*74*). State health departments or their bona fide agents manage the state violent death reporting systems and serve as the point of contact to collect information from the major data sources.

NVDRS obtains data from multiple complementary data sources, including death certificates, medical examiner and coroner records, law enforcement reports, and toxicology reports. Individual-level data include manner of death, injury mechanism, whether the person involved in an incident was a victim, and circumstances, which are defined as the precipitating events that contributed to the fatal injury. Numerous types of circumstance data are collected for suicide in NVDRS, including factors such as mental health history and status (e.g., current depressed mood, current mental health problems, current treatment for mental health problems, whether treatment has ever been received for mental health problems, and history of suicide attempts), whether a decedent disclosed the intent to die by suicide, interpersonal conflicts, alcohol or other substance use, other addictions, and criminal acts. Short narratives are also written by abstractors, which provide more details about the incident, summarizing the sequence of events from the perspectives of the medical examiner or coroner and law enforcement. Aggregate counts, percentages, and crude rates are available for all deaths by abstractor-assigned manner of death. Because no sampling is involved, all identified violent deaths in states that meet the reporting criteria are included. Data for persons aged 10–19 years in 18 states during 2014–2018 are included in this report (Alaska, Colorado, Georgia, Kentucky, Maryland, Massachusetts, Minnesota, New Jersey, New Mexico, North Carolina, Ohio, Oklahoma, Oregon, Rhode Island, South Carolina, Utah, Virginia, and Wisconsin). NVDRS data are publicly accessible through CDC’s Web-based Injury Statistics Query and Reporting System (WISQARS; https://www.cdc.gov/injury/wisqars/nvdrs.html) (*41*). Deidentified case-level data are also available by formal request to eligible researchers via access to the NVDRS Restricted Access Database (https://www.cdc.gov/violenceprevention/datasources/nvdrs/dataaccess.html).

#### National Vital Statistics System

Vital statistics mortality data from NVSS are a fundamental source of demographic, geographic, and cause-of-death information in the United States (https://www.cdc.gov/nchs/nvss/index.htm). The data are used to present characteristics of persons who have died in the United States, understand leading causes of death, determine life expectancy, and compare the U.S. mortality data with those in other countries. The NVSS mortality data include information on all deaths occurring within the United States annually and have been collected since 1900. Mortality data are collected from information reported on death certificates, which are completed by funeral directors (the demographic portion) and the attending physicians, medical examiners, or coroners (the medical portion). National data for vital statistics are provided through contracts between CDC’s NCHS and state vital registration systems that are legally responsible for the registration of deaths. Information on demographics, geographic details, and cause of death is provided for all decedents. State- and county-level information is available by place of occurrence as well as place of residence. Causes of death, including suicide, are processed in accordance with the *International Classification of Diseases, Tenth Revision* (ICD-10). Data are available in various formats, including reports, downloadable data files (https://www.cdc.gov/nchs/data_access/vitalstatsonline.htm), restricted use data files (https://www.cdc.gov/nchs/nvss/nvss-restricted-data.htm), and online query systems (https://wonder.cdc.gov). Data for persons aged 10–19 years for 2018–2019 are included in this report.

#### School-Associated Violent Death Surveillance System

SAVD-SS is maintained by CDC and was designed to monitor incidents of lethal violence, including suicides, homicides, and legal intervention deaths, that occur in and around school settings (https://www.cdc.gov/violenceprevention/youthviolence/schoolviolence/SAVD.html). Data on school-associated violent deaths (SAVDs) are available for 1994–2018. Incidents are identified through a systematic media scan of computerized newspaper and broadcast media databases. SAVD-SS cases include incidents in which a death occurred 1) on the campus of a functioning U.S. public or private primary or secondary school, 2) while the victim was on the way to or from regular sessions at such a school, or 3) while the victim was attending or traveling to or from an official school-sponsored event. Identified incidents are confirmed with local law enforcement or school officials familiar with the incident, and law enforcement reports are collected when possible. Incident, victim, and perpetrator characteristics (e.g., incident location, cause and manner of death, circumstances precipitating the incident, and firearm-related information for incidents involving firearms) are coded from law enforcement reports, interviews with law enforcement or school officials familiar with each incident, or articles published in the media when a reliable source is cited (i.e., a law enforcement or school official or judicial proceedings regarding the incident). SAVD-SS also collects information about whether the victim and perpetrator had a suspected or diagnosed mental health condition, experienced suicidal ideation or had a history of suicide attempts, were using alcohol or substances at the time of death or regularly used alcohol or substances, and had been victimized or perpetrated violence in the past.

Because SAVDs are relatively rare and SAVD-SS includes personally identifiable information, data are not made publicly available; however, aggregate data are reported every school year (July 1–June 1) in the U.S. Department of Education’s Annual Indicators of School Crime and Safety Report. Additional information about SAVD-SS is available (https://www.cdc.gov/violenceprevention/youthviolence/schoolviolence/SAVD.html).

This report provides data on school-associated suicides among persons aged 10–18 years during 2013–2018. Beginning in January 2021, SAVD-SS data from 2021 and later will be collected through CDC’s NVDRS and will be publicly available in 2023.

#### National Youth Risk Behavior Survey

The Youth Risk Behavior Surveillance System (YRBSS) monitors health behaviors and experiences among U.S. high school students that contribute to the leading causes of mortality, morbidity, and social problems among youths and adults (https://www.cdc.gov/yrbss). The system includes a national YRBS, conducted by CDC, and separate state, tribal, territorial, and local school district school-based YRBSs. National YRBS data and data for many state, territorial, and local school districts are publicly available (https://www.cdc.gov/yrbss).

Since 1991, the national YRBS has been conducted biennially, using independent, three-stage cluster sample designs to produce representative samples of public and private high school students in grades 9–12 (primarily aged 14–18 years) in the 50 states and DC. Student participation in the YRBS is anonymous and voluntary, and local parental permission procedures are used. Survey participants complete a self-administered pencil-and-paper questionnaire during a regular class period and record their responses on a computer-scannable answer sheet. In 2019, the number of students in the sample was 13,677, the school response rate was 75%, the student response rate was 80%, and the overall response rate (the product of the school and student response rates) was 60%.

Indicators of mental health included in the national YRBS include persistent feelings of sadness or hopelessness and suicide-related behaviors. To assess persistent feelings of sadness or hopelessness, students were asked if during the past 12 months, they had ever felt so sad or hopeless almost every day for ≥2 weeks in a row that they stopped doing some usual activities. To assess suicidal ideation and suicide attempts, students were asked if during the past 12 months they ever seriously considered attempting suicide, whether they made a plan about how they would attempt suicide, the number of times they had actually attempted suicide, and whether they had made a suicide attempt that resulted in an injury, poisoning, or an overdose that had to be treated by a physician or nurse. Although not included in this report, YRBS also monitors the prevalence of alcohol, tobacco, and other drug use; however, the questions are not designed to identify drug use disorders. These data and the prevalence of other health risk behaviors and experiences examined by the national YRBS and many state, territorial, and local school district YRBSs are publicly available (www.cdc.gov/yrbss).

### Analysis

Similar indicators from different surveillance systems are grouped together and described (Tables 3–13), using data from the most recent years available, as defined in previous sections. Weighted prevalence estimates and 95% CIs were calculated overall and by sociodemographic characteristics for the population represented by each surveillance system. No statistical tests were conducted. The 95% CIs were compared, and estimates with nonoverlapping CIs were considered to be significantly different. This is an inherently conservative approach to the identification of differences between estimates and might not have detected certain significant differences that would have been identified using other methods. Most estimates included children and adolescents aged 3–17 years, although certain surveillance systems were limited to data on children and adolescents with a narrower age range (ADDM: 8 years; NSDUH: 12–17 years; and YRBS: high school students, primarily aged 14–18 years); indicators related to suicide include persons aged 10–19 years (NVDRS and NVSS) and 10–18 years (SAVD-SS), and NSCH included certain positive mental health indicators for children as young as age 6 months. Indicators of mental health services are presented as the estimated percentage of all children who received a particular mental health service (e.g., consultations or medication), rather than the percentage of children with a diagnosed disorder, to allow for more comparable estimates across surveillance systems. Mental health services data are included from surveillance systems that also include prevalence of disorders. (Data sources that only included treatment data without other indicators of mental health were not included.) Estimates by sociodemographic characteristics were calculated by age group, sex, race and ethnicity, household federal poverty level (FPL), highest level of education among parents, health insurance status, and geographic classification (urban/suburban versus rural) as available and applicable within each surveillance system. Subgroup estimates by race and ethnicity were calculated for non-Hispanic White (White), non-Hispanic Black (Black), Hispanic, and non-Hispanic Asian (Asian) children; for a limited number of indicators with sufficient sample size, estimates are presented for non-Hispanic American Indian or Alaska Native (AI/AN) children and non-Hispanic Native Hawaiian or other Pacific Islander (NH/OPI) children. Estimates for children in other racial and ethnic groups were not calculated because the sample sizes were too small to produce stable estimates and are not presented in the tables. Estimates by health insurance status are presented for children with any type of public health insurance, children with any private health insurance, and children with no health insurance. Children with both public and private health insurance were represented in both subcategories. The information used to indicate geographic classification differed among surveillance systems*; the urban/suburban subgroup consists of persons living in large metropolitan areas with a metropolitan statistical area (MSA) population of ≥1 million and suburban areas including medium and small metropolitan areas with MSA populations of <1 million; the rural subgroup consists of those living in all other areas.

**TABLE 3:** Estimated number and prevalence of persons aged 0–19 years with certain mental disorders and indicators of mental health*,* by surveillance system, year of data collection, and age group — United States, 2013–2019

Disorder or indicator	Surveillance system and data collection years	Age group	No. of persons surveyed	Weighted prevalence (%)*	Weighted no.^†^ of children with reported indicator
**ADHD**					
Ever (parent ever told by health care provider child had ADHD)	NSCH 2016‒2019	3‒17 yrs	114,476	9.8	5,964,000
	NHIS 2017‒2018	3‒17 yrs	14,316	9.6	5,952,000
Current (parent reported child currently had ADHD)	NSCH 2016‒2019	3‒17 yrs	114,476	8.7	5,319,000
	NHIS 2017‒2018	3‒17 yrs	14,292	8.2	5,043,000
**Behavioral or conduct problems**					
Ever (parent ever told by health care provider child had behavioral or conduct problems)	NSCH 2016‒2019	3‒17 yrs	114,476	8.9	5,478,000
Current (parent reported child currently had behavioral or conduct problems)	NSCH 2016‒2019	3‒17 yrs	114,476	7.0	4,265,000
**Depression**					
Ever depression (parent ever told by health care provider child had depression)	NSCH 2016‒2019	3‒17 yrs	114,476	4.4	2,729,000
Current depression (parent reported child currently had depression)	NSCH 2016‒2019	3‒17 yrs	114,476	3.4	2,093,000
Ever had MDE (self-report)^§^	NSDUH 2018‒2019	12‒17 yrs	33,678	20.9	5,068,000
Past year MDE (self-report)^§^	NSDUH 2018‒2019	12‒17 yrs	33,678	15.1	3,633,000
Current depression (self-reported depression during past 2 wks; score of ≥10 on PHQ-9 depression screener)	NHANES 2013‒2018	12‒17 yrs	2,771	5.8	2,168,000
Sadness or hopelessness (self-reported feeling so sad or hopeless almost every day for ≥2 wks in a row that they stopped doing some usual activities)	National YRBS 2019	~14‒18 yrs^¶^	13,677	36.7	6,145,000
**Anxiety**					
Ever (parent ever told by health care provider child had anxiety problems)	NSCH 2016‒2019	3‒17 yrs	114,476	9.4	5,769,000
Current (parent reported child currently had anxiety)	NSCH 2016‒2019	3‒17 yrs	114,476	7.8	4,768,000
**Autism spectrum disorder**					
Ever (parent ever told by health care provider child had ASD)	NSCH 2016‒2019	3‒17 yrs	114,476	3.1	1,881,000
	NHIS 2017‒2018	3‒17 yrs	14,324	2.4	1,468,000
Current (parent reported child currently had ASD)	NSCH 2016‒2019	3‒17 yrs	114,476	2.9	1,766,000
	NHIS 2017‒2018	3‒17 yrs	14,320	2.0	1,266,000
Met ASD surveillance case definition**	ADDM Network 2016	8 yrs	275,419^††^	1.9	NA^§§^
**Tourette syndrome**					
Ever (parent ever told by health care provider child had Tourette syndrome)	NSCH 2016‒2019	3‒17 yrs	114,476	0.3	174,000
Current (parent reported child currently had Tourette syndrome)	NSCH 2016‒2019	3‒17 yrs	114,476	0.2	136,000
**Substance use disorder** ^¶¶^					
Past year substance use disorder	NSDUH 2018‒2019	12‒17 yrs	33,678	4.1	1,017,000
Past year alcohol use disorder	NSDUH 2018‒2019	12‒17 yrs	33,678	1.6	407,000
Past year illicit drug use disorder	NSDUH 2018‒2019	12‒17 yrs	33,678	3.2	788,000
**Suicidality**					
Seriously considered attempting suicide (during 12 mos before survey)	YRBS 2019	~14‒18 yrs^¶^	13,677	18.8	3,148,000
Made a suicide plan (during 12 mos before survey)	YRBS 2019	~14‒18 yrs^¶^	13,677	15.7	2,629,000
Attempted suicide ≥1 time (during 12 mos before survey)	YRBS 2019	~14‒18 yrs^¶^	13,677	8.9	1,490,000
Made a suicide attempt ≥1 time (during 12 mos before survey) that resulted in injury, poisoning, or overdose that had to be treated by physician or nurse	YRBS 2019	~14‒18 yrs^¶^	13,677	2.5	419,000
No. of suicides	NVSS 2018‒2019	10‒19 yrs	5,744 deaths	6.9/100,000	NA
No. of suicides	NVDRS*** 2014‒2018	10‒19 yrs	4,903 deaths	7.0/100,000	NA
**Mental health services**					
Mental health treatment, professional^†††^	NSCH 2016‒2019	3‒17 yrs	114,476	10.1	6,197,000
Mental health consultation, professional^§§§^	NHIS 2017‒2018	3‒17 yrs	14,287	9.6	5,939,000
Mental health consultation, general physician^¶¶¶^	NHIS 2017‒2018	3‒17 yrs	14,253	5.2	3,222,000
Mental health services received (specialty and nonspecialty)	NSDUH 2018‒2019	12‒17 yrs	33,678	25.9	6,358,000
Mental health consultation, professional****	NHANES 2013–2018	4‒17 yrs	8,071	9.8	5,664,000
Past year medication for mental health problems^††††^	NSCH 2016‒2019	3‒17 yrs	114,476	7.8	4,724,000
Current use of psychotherapeutic agents in past 30 days for mental health problems	NHANES 2013–2018	3‒17 yrs	8,637	6.6	4,082,000
**Positive indicators of child mental health **					
Affectionate (usually or always affectionate and tender with parent)	NSCH 2018‒2019	6 mos‒5 yrs	15,844	97.3	21,055,000
Resilient (usually or always bounces back quickly when things do not go their way)	NSCH 2018‒2019	6 mos‒5 yrs	15,844	89.8	19,457,000
Positivity (usually or always smiles and laughs a lot)	NSCH 2018‒2019	6 mos‒5 yrs	15,844	99.0	21,451,000
Curious (usually or always shows interest and curiosity in learning new things)	NSCH 2018‒2019	6 mos‒17 yrs	59,057	91.3	64,912,000
Persistent (usually or always works to finish tasks)	NSCH 2018‒2019	6‒17 yrs	43,213	84.5	40,457,000
Self**-**control (usually or always stays calm and in control when faced with a challenge)	NSCH 2018‒2019	6‒17 yrs	43,213	76.8	37,757,000

**Abbreviations**: ADDM = Autism and Developmental Disabilities Monitoring; ADHD = attention-deficit hyperactivity disorder; ASD = autism spectrum disorder; DSM-IV = *Diagnostic and Statistical Manual of Mental Disorders*, *4th Edition*, MDE = major depressive episode; NA = not applicable; NHANES = National Health and Nutrition Examination Survey; NHIS = National Health Interview Survey; NSCH = National Survey of Children’s Health; NSDUH = National Survey on Drug Use and Health; NVDRS = National Violent Death Reporting System; NVSS = National Vital Statistics System; PHQ-9 = nine-item Patient Health Questionnaire; YRBS = Youth Risk Behavior Survey.

* All estimates are weighted, except for 1) the prevalence of ASD from ADDM, calculated as number of cases identified divided by number of children living in catchment area and 2) suicide rates for NVSS and NVDRS, calculated as number of suicides per 100,000 persons aged 10‒19 years

^†^ *NSCH:* weighted using sample child weights that adjust for sampling probability, nonresponse, and raking adjustments. Raking adjustments used population controls from the 2015–2018 Census Bureau’s American Community Survey. *NHIS:* weighted using sample child weights that adjust for the probability of selection, nonresponse, and poststratification. Poststratification adjustments for this report use population estimates derived from the 2010 Census by the Census Bureau. *NHANES:* weighted using interview and examination sample weights, adjusted for the probability of selection, non-response, and calibration. Calibration adjustments and population estimates are based on age-specific Census Bureau American Community Surveys from 2013, 2015, and 2017. *NSDUH:* individual observations weighted so that the weighted sample represents the civilian, noninstitutionalized population in the United States. The person-level weights in NSDUH are calibrated by adjusting for nonresponse and poststratifying to known population estimates (or control totals) obtained from the Census Bureau. *YRBS:* estimates multiplied by 16,745,000, the number of public and private high school students in the United States in 2019.

^§^ Ever MDE: reported at least five or more of nine symptoms nearly every day in the same 2-week period during their lifetime, in which at least one of the symptoms was depressed mood or loss of interest or pleasure in daily activities; MDE in past year: 1) had ever had an MDE, as well as 2) had a period of time in the past 12 months when they felt depressed or lost interest or pleasure in daily activities for ≥2 weeks and 3) during this period of ≥2 weeks, they had some of the other problems they reported associated with ever having had an MDE.

^¶^ Survey participants were public and private high school students in grades 9–12 (i.e., primarily aged 14–18 years).

** Case definition based on *Diagnostic and Statistical Manual of Mental Disorders*, *5th Edition* criteria for autism spectrum disorder. Clinicians applied the case definition through a review information systematically collected from developmental evaluations completed by medical and educational service providers in the community.

^††^ Population denominator for ADDM catchment areas.

^§§^ ADDM estimates are not weighted or intended to be extrapolated to the entire U.S. population.

^¶¶^ Substance use disorder: meets DSM-IV criteria for either dependence or abuse for one or more illicit drugs or alcohol; alcohol use disorder: meets criteria for either alcohol dependence or abuse; illicit drug use disorder: meets criteria for either dependence or abuse for one or more illicit drugs.

*** States (n = 18) included Alaska, Colorado, Georgia, Kentucky, Maryland, Massachusetts, Michigan, New Jersey, New Mexico, North Carolina, Ohio, Oklahoma, Oregon, Rhode Island, South Carolina, Utah, Virginia, and Wisconsin.

^†††^ “During the past 12 months, has this child received any treatment or counseling from a mental health professional?”

^§§§^ “During the past 12 months, have you seen or talked to...a mental health professional such as a psychiatrist, psychologist, psychiatric nurse, or clinical social worker...about child's health?”

^¶¶¶^ “Did you see or talk to this general doctor because of an emotional or behavioral problem that [child] may have?”

**** “During the past 12 months, have you seen or talked to...a mental health professional such as a psychiatrist, psychologist, psychiatric nurse, or clinical social worker...about child's health?”

^††††^ “During the past 12 months, has this child taken any medication because of difficulties with his or her emotions, concentration, or behavior?”

**TABLE 13:** Weighted prevalence estimates of positive indicators of mental health among children and adolescents aged 6 months–17 years, by sociodemographic characteristics — National Survey of Children’s Health, United States, 2018‒2019

Characteristic	Affection*	Resilience^†^	Positivity^§^	Curiosity^¶^	Persistence**	Self-control^††^
**Age group**	6 mos–5 yrs	6 mos–5 yrs	6 mos–5 yrs	6 mos–17 yrs	6–17 yrs	6–17 yrs
**Sample size (no.)**	15,844	15,844	15,844	59,057	43,213	43,213
**Total**	**97.3 (96.7‒97.8)**	**89.8 (88.7‒90.8)**	**99.0 (98.5‒99.3)**	**91.3 (90.8‒91.8)**	**84.5 (83.7‒85.2)**	**76.8 (76.0‒77.7)**
**Age group (yrs)**						
3–5	97.0 (96.3‒97.6)	87.9 (86.2‒89.4)	98.7 (97.9‒99.1)	93.9 (92.7‒93.8)	NA	NA
6–11	NA	NA	NA	93.0 (92.2‒93.8)	84.2 (83.1‒85.3)	73.8 (72.6‒75.1)
12–17	NA	NA	NA	86.5 (85.5‒87.5)	84.7 (83.7‒85.7)	79.8 (78.6‒80.9)
**Sex**						
Male	97.0 (96.2‒97.7)	88.6 (86.9‒90.1)	98.7 (98.0‒99.2)	89.9 (89.2‒90.6)	81.2 (80.1‒82.3)	74.3 (73.1‒75.5)
Female	97.5 (96.6‒98.2)	91.1 (89.6‒92.4)	99.2 (98.6‒99.6)	92.8 (92.0‒93.4)	87.8 (86.8‒88.8)	79.5 (78.2‒80.6)
**Race/Ethnicity** ^§§^						
Hispanic	96.8 (94.8‒98.0)	85.5 (82.0‒88.4)	98.9 (97.5‒99.5)	89.4 (87.8‒90.8)	84.3 (82.0‒86.3)	76.5 (74.0‒78.9)
Black non-Hispanic	97.7 (96.3‒98.6)	85.2 (80.9‒88.6)	97.8 (95.2‒99.0)	89.9 (88.5‒91.2)	80.4 (78.0‒82.5)	74.3 (71.8‒76.7)
White, non-Hispanic	97.5 (96.8‒98.0)	93.7 (92.7‒94.6)	99.3 (99.0‒99.5)	92.7 (92.3‒93.2)	85.5 (84.7‒86.2)	77.1 (76.2‒77.9)
Asian, non-Hispanic	96.7 (94.2‒98.2)	79.3 (73.1‒84.3)	99.5 (98.5‒99.8)	91.7 (89.3‒93.6)	87.8 (84.0‒90.9)	85.7 (82.6‒88.3)
**FPL** ^¶¶^						
≤100% FPL	95.4 (93.0‒97.0)	83.1 (79.3‒86.3)	98.6 (97.5‒99.2)	86.3 (84.7‒87.8)	77.3 (75.0‒79.5)	69.4 (66.8‒71.9)
>100% to ≤200% FPL	96.4 (94.9‒97.5)	88.1 (85.1‒90.6)	99.0 (98.3‒99.5)	89.5 (88.1‒90.8)	81.7 (79.7‒83.6)	74.1 (72.0‒76.1)
>200% FPL	98.2 (97.8‒98.6)	92.7 (91.7‒93.6)	99.0 (98.4‒99.4)	93.6 (93.1‒94.1)	87.7 (86.9‒88.4)	80.3 (79.4‒81.2)
Highest level of parent education***						
Less than high school	97.3 (92.5‒99.1)	82.5 (74.5‒88.4)	97.4 (92.1‒99.2)	84.9 (81.7‒87.6)	79.7 (75.6‒83.3)	72.9 (68.4‒77.1)
High school graduate	95.0 (92.6‒96.6)	84.2 (80.0‒87.6)	97.7 (95.8‒98.7)	87.6 (86.1‒89.0)	79.7 (77.6‒81.6)	72.7 (70.5‒74.8)
More than high school	97.8 (97.3‒98.2)	91.9 (90.9‒92.7)	99.4 (99.2‒99.6)	93.2 (92.7‒93.6)	86.4 (85.7‒87.1)	78.6 (77.7‒79.4)
**Health insurance** ^†††^						
Yes						
Any public	95.9 (94.5‒96.9)	85.9 (83.5‒87.9)	98.7 (97.9‒99.2)	87.7 (86.6‒88.8)	77.0 (75.3‒78.7)	68.6 (66.8‒70.4)
Any private	97.8 (97.0‒98.4)	92.0 (90.7‒93.0)	99.4 (99.0‒99.6)	93.6 (93.1‒94.1)	87.6 (86.8‒88.3)	80.3 (79.4‒81.2)
No insurance	96.5 (93.8‒98.1)	88.4 (80.5‒93.4)	98.1 (95.8‒99.2)	87.5 (84.5‒89.9)	82.2 (78.5‒85.4)	76.3 (72.1‒80.1)
**Geographic classification** ^§§§^						
Urban/Suburban	97.5 (96.8‒98.1)	89.7 (88.2‒91.0)	98.9 (98.4‒99.3)	91.5 (90.9‒92.1)	84.7 (83.7‒85.6)	77.5 (76.4‒78.5)
Rural	96.2 (94.3‒97.5)	92.1 (89.6‒94.1)	99.2 (98.3‒99.6)	89.8 (88.6‒91.0)	82.5 (80.6‒84.2)	73.4 (71.3‒75.4)

**Abbreviations**: FPL = federal poverty level; NA = not available; NSCH = National Survey of Children’s Health.

* “This child is affectionate and tender with you” (usually or always).

^†^ “This child bounces back quickly when things do not go his or her way” (usually or always).

^§^ “This child smiles and laughs a lot” (usually or always).

^¶^ “This child shows interest and curiosity in learning new things” (usually or always).

** “This child works to finish tasks he or she starts” (usually or always).

^††^ “This child stays calm and in control when faced with a challenge” (usually or always).

^§§^ Estimates exclude other race and ethnicity groups that did not have a large enough sample size to produce stable estimates.

^¶¶^ FPL is based on family income and family size using the Census Bureau’s poverty thresholds for the previous calendar year. Imputed income files were used to impute family income when it was not provided and family size was imputed using other information about the household when the number of family members was not provided (https://www.census.gov/topics/income-poverty/poverty/guidance/poverty-measures.html).

*** The highest level of parent education is based on the highest education level among up to two adults who were identified as primary caregivers in the survey.

^†††^ Private included any insurance from an employer or union, directly purchased, TRICARE or other military health care, or the Affordable Care Act; coverage from any government assistance plan was considered public, including Medicaid or other state-sponsored health plans including the Children’s Health Insurance Program. Respondents who indicated both public and private insurance coverage were represented in both subcategories.

^§§§^ Method for determining geographic classification for NSCH was based on the 2010 Office of Management and Budget metropolitan and micropolitan statistical areas standards (https://www.govinfo.gov/content/pkg/FR-2010-06-28/pdf/2010-15605.pdf). Urban/suburban includes metropolitan statistical areas associated with at least one urbanized area of at least 50,000 population; rural was defined as counties that were not part of a metropolitan statistical area.

## Results

Descriptions of mental disorders and mental health indicators are included in this report, as well as prevalence estimates of the disorders and indicators (including rates of suicide) among children and adolescents available from national surveillance systems. State prevalence estimates and rates are provided when available. Overall prevalence estimates, including weighted population estimates of the number of U.S. children and adolescents with each disorder or indicator, are presented (Table 3); rates per 100,000 are presented for suicide. NSCH 2016–2019 data indicated that ADHD (9.8%) and anxiety (9.4%) were the most common mental disorders among U.S. children and adolescents aged 3–17 years; NHIS 2017–2018 data indicated an estimate of 9.6% for ADHD. Among adolescents, data from NSDUH 2018–2019 data indicated that 15.1% of adolescents aged 12–17 years had an MDE in the past year, and YRBS 2019 data indicated that 36.7% of high school students aged primarily 14–18 years experienced persistent feelings of sadness or hopelessness during the past year. In addition, YRBS data indicated that during the past year, 18.8% of high school students had seriously considered attempting suicide, and 8.9% had attempted suicide one or more times. Approximately 10% (9.6%–10.1% across NSCH 2016–2019 survey years, NHIS 2017–2018 survey years, and NHANES 2013–2018 survey years) of U.S. children and adolescents aged 3–17 years received mental health services from a mental health professional in the past year, and 7.8% had taken medication because of difficulties with emotions, concentration, or behavior in the past year according to NSCH data. Approximately one fourth (25.9%) of adolescents aged 12–17 years reported receiving mental health services in the past year, according to self-reported responses from NSDUH. Positive indicators of mental health were reported for at least three fourths of children aged 6 months–17 years.

### Attention-Deficit/Hyperactivity Disorder 

ADHD is a neurodevelopmental disorder characterized by symptoms of inattention, hyperactivity, and impulsivity that are present in multiple contexts, such as at home, at school, or with friends, and cause significant impairment (*1*,*75*). On the basis of the predominant symptoms at the time of diagnosis, children can be classified into one of the three following categories: inattentive, hyperactive/impulsive, or combined (*75*). However, symptoms can change over time (*76*). ADHD is associated with a substantial risk for educational and occupational failure, criminality, social disability, substance use, other mental disorders, injuries, illness, and lower life expectancy (*77*–*79*). Both the NSCH and NHIS questionnaires asked parents whether a health care provider had ever told them that their child had ADHD (ever ADHD) and whether the child currently had this condition (current ADHD).

Data from the 2016–2019 survey years of NSCH indicate that among children and adolescents aged 3–17 years, parents reported that 9.8% had ever received a diagnosis of ADHD, and 8.7% currently had ADHD (Table 4). These prevalence estimates were very similar to the results from the 2017 and 2018 survey years of NHIS (Table 4), which assessed the same age range using the same survey item, with prevalence estimates of 9.6% and 8.2%, respectively. State prevalence estimates from NSCH of ever ADHD among children and adolescents aged 3–17 years ranged from 6.1% in California to 16.3% in Louisiana; prevalence estimates of current ADHD ranged from 5.3% in California to 14.4% in Mississippi (Supplementary Tables 1 and 2; https://stacks.cdc.gov/view/cdc/113924).

**TABLE 4:** Weighted prevalence estimates of attention-deficit/hyperactivity disorder among children and adolescents aged 3–17 years, by sociodemographic characteristics and surveillance system — United States, 2016–2019

Characteristic	Ever had ADHD		Current ADHD	
	NSCH 2016–2019	NHIS 2017–2018	NSCH 2016–2019	NHIS 2017–2018
	% (95% CI)	% (95% CI)	% (95% CI)	% (95% CI)
**Age group (yrs)**	3–17	3–17	3–17	3–17
**Sample size (no.)**	114,476	14,316	114,476	14,292
**Total**	**9.8 (9.4‒10.1)**	**9.6 (9.0‒10.2)**	**8.7 (8.4‒9.1)**	**8.2 (7.6‒8.7)**
**Age group (yrs)**				
3–5	2.2 (1.8‒2.8)	1.8 (1.2‒2.6)	2.0 (1.6‒2.5)	1.6 (1.1‒2.3)
6–11	10.0 (9.4‒10.6)	9.7 (8.8‒10.7)	9.3 (8.7‒9.8)	8.7 (7.8‒9.6)
12–17	13.2 (12.6‒13.8)	13.4 (12.4‒14.4)	11.5 (10.9‒12.0)	10.8 (10.0‒11.8)
**Sex**				
Male	13.3 (12.8‒13.9)	12.9 (12.0‒13.8)	11.9 (11.3‒12.4)	11.0 (10.2‒12.0)
Female	6.1 (5.7‒6.5)	6.2 (5.6‒6.9)	5.5 (5.1‒5.9)	5.2 (4.6‒5.8)
Race/Ethnicity*				
Hispanic	7.5 (6.7‒8.5)	7.0 (5.9‒8.2)	6.6 (5.9‒7.5)	5.4 (4.5‒6.5)
Black, non-Hispanic	12.0 (10.8‒13.4)	11.4 (9.7‒13.5)	10.5 (9.4‒11.8)	9.9 (8.1‒11.9)
White, non-Hispanic	10.9 (10.6‒11.3)	10.9 (10.1‒11.7)	9.9 (9.5‒10.3)	9.4 (8.7‒10.2)
Asian, non-Hispanic	2.6 (2.0‒3.3)	2.1 (1.3‒3.3)	2.2 (1.7‒2.9)	1.6 (0.9‒2.6)
**FPL** ^†^				
≤100% FPL	11.2 (10.3‒12.2)	11.5 (10.0‒13.2)	10.2 (9.3‒11.1)	10.0 (8.6‒11.6)
>100% to ≤ 200% FPL	10.5 (9.6‒11.5)	11.2 (9.9‒12.7)	9.3 (8.4‒10.1)	9.6 (8.4‒11.0)
>200% FPL	9.0 (8.6‒9.4)	8.5 (7.8‒9.2)	8.0 (7.7‒8.4)	7.1 (6.4‒7.8)
**Highest level of parent education^§^**				
Less than high school	9.2 (7.6‒11.2)	8.2 (6.6‒10.2)	8.0 (6.5‒9.9)	7.1 (5.7‒9.0)
High school graduate	11.0 (10.1‒12.0)	10.8 (9.3‒12.4)	10.0 (9.1‒11.0)	8.9 (7.6‒10.4)
More than high school	9.5 (9.2‒9.8)	9.2 (8.5‒9.9)	8.5 (8.2‒8.8)	7.8 (7.1‒8.5)
**Health insurance^¶^**				
Yes				
Any public	13.2 (12.4‒14.0)	12.4 (11.4‒13.5)	12.1 (11.4‒12.8)	10.7 (9.7‒11.7)
Any private	8.7 (8.3‒9.1)	8.3 (7.3‒9.3)	7.7 (7.4‒8.1)	7.2 (6.3‒8.2)
No insurance	7.1 (5.9‒8.7)	6.1 (4.3‒8.6)	5.8 (4.8‒7.1)	4.7 (3.2‒7.0)
Geographic classification**				
Urban/Suburban	9.5 (9.0‒9.9)	9.3 (8.7‒10.0)	8.4 (8.0‒8.8)	7.9 (7.3‒8.5)
Rural	12.0 (11.1‒12.9)	12.0 (10.4‒13.7)	10.7 (9.9‒11.6)	10.0 (8.6‒11.6)

**Abbreviations:** ADHD = attention-deficit/hyperactivity disorder; FPL = federal poverty level; NHIS = National Health Interview Survey; NSCH = National Survey of Children’s Health.

* Estimates exclude other race and ethnicity groups that did not have a large enough sample size to produce stable estimates.

^†^ FPL is based on family income and family size using the Census Bureau’s poverty thresholds for the previous calendar year. Imputed income files were used to impute family income when it was not provided, and for NSCH, family size was imputed using other information about the household when the number of family members was not provided (https://www.census.gov/topics/income-poverty/poverty/guidance/poverty-measures.html).

^§^ The highest level of parent education is based on the highest education level among up to two adults who were identified as primary caregivers in the survey.

^¶^ Private included any insurance from an employer or union, directly purchased, TRICARE or other military health care, or the Affordable Care Act; coverage from any government assistance plan was considered public, including Medicaid or other state-sponsored health plans including the Children’s Health Insurance Program. Respondents who indicated both public and private insurance coverage were represented in both subcategories.

** Methods for determining geographic classification differed by survey. *NSCH:* 2010 Office of Management and Budget metropolitan and micropolitan statistical areas standards (https://www.govinfo.gov/content/pkg/FR-2010-06-28/pdf/2010-15605.pdf); urban/suburban includes metropolitan statistical areas associated with at least one urbanized area of at least 50,000 population; rural was defined as counties that were not part of a metropolitan statistical area. *NHIS:* 2013 National Center for Health Statistics urban/rural classification (https://www.cdc.gov/nchs/data/series/sr_02/sr02_166.pdf); urban/suburban includes large metropolitan, medium metropolitan, and small metropolitan areas, whereas rural includes nonmetropolitan areas with <50,000 population.

Data from both NSCH and NHIS indicated that the prevalence of ADHD was higher among older age groups, and boys had approximately double the prevalence of ADHD diagnoses compared with girls. Among racial and ethnic groups, Hispanic and Asian children had the lowest prevalence, whereas Black and White children had the highest prevalence. Certain socioeconomic indicators, specifically, being in the lowest household income category (≤200% FPL) and having public health insurance, were associated with higher prevalence of ADHD. Parent education level was also associated with ADHD prevalence, based on NSCH data; children who had a parent with more than a high school education had a lower ADHD prevalence than those who had a parent with a high school education. The prevalence of ADHD among children whose parents had less than a high school education was similar to that among children who had a parent with more than a high school education; NHIS estimates of ADHD did not differ by parent education level. When examining prevalence by geographic classification, both NSCH and NHIS data indicated that a higher proportion of children in rural areas had ADHD compared with children in urban or suburban areas.

### Behavioral and Conduct Problems

Problems with behavior and conduct (i.e., behavior problems) among children and adolescents are associated with risks for long-term problems, including educational and occupational failure, substance use, mental disorders, injury, violence, delinquency, and lower life expectancy (*77*,*80*). Disruptive behaviors that cause conflict between a child and family members, peers, and authority figures can be diagnosed as disorders, such as oppositional defiant disorder and conduct disorder (*1*). Oppositional, inappropriate, negative, or defiant behavior is often found in younger children (*1*). Conduct problems are often found in older children and are characterized by behavior focused on ignoring the rights of others and violating social norms and rules (*1*). NSCH was the only survey with a question that asked parents if a health care provider or educator (including teachers and school nurses) had ever told them that their child had behavioral or conduct problems, followed by a question on whether the child currently had this problem.

Data from the 2016–2019 survey years of NSCH indicated that 8.9% of children and adolescents aged 3–17 years had ever received a diagnosis of behavior problems, and 7.0% had behavior problems at the time of the survey (Table 5). State prevalence estimates of ever having had behavior problems among children and adolescents aged 3–17 years ranged from 6.4% in California to 13.1% in Louisiana; estimates of current behavior problems ranged from 4.1% in California to 10.9% in Kentucky (Supplementary Tables 1 and 2; https://stacks.cdc.gov/view/cdc/113924).

**TABLE 5:** Weighted prevalence estimates of behavioral or conduct problems among children and adolescents aged 3–17 years, by sociodemographic characteristics — National Survey of Children's Health, United States, 2016–2019

Characteristic	Ever had behavioral or conduct problems	Current behavioral or conduct problems
	% (95% CI)	% (95% CI)
**Age group (yrs)**	3–17	3–17
**Sample size (no.)**	114,476	114,476
**Total**	**8.9 (8.6‒9.3)**	**7.0 (6.6‒7.3)**
**Age group (yrs)**		
3–5	5.0 (4.4–5.6)	3.8 (3.4–4.4)
6–11	10.4 (9.8–11.1)	8.6 (8.1–9.2)
12–17	9.3 (8.8–9.8)	6.8 (6.3–7.3)
**Sex**		
Male	12.2 (11.6–12.8)	9.4 (8.9–9.9)
Female	5.5 (5.1–5.9)	4.4 (4.1–4.8)
Race/Ethnicity*		
Hispanic	7.2 (6.4–8.1)	5.6 (4.9–6.4)
Black, non-Hispanic	13.1 (11.8–14.5)	10.1 (9.0–11.4)
White, non-Hispanic	8.9 (8.6–9.3)	7.0 (6.7–7.4)
Asian, non-Hispanic	3.4 (2.6‒4.3)	2.5 (1.9‒3.3)
**FPL^†^**		
≤100% FPL	12.4 (11.4‒13.6)	10.3 (9.4‒11.4)
>100% to ≤200% FPL	9.5 (8.8‒10.4)	7.6 (7.0‒8.3)
>200% FPL	7.4 (7.1‒7.8)	5.5 (5.2‒5.8)
**Highest level of parent education^§^**		
Less than high school	8.9 (7.3‒10.7)	7.1 (5.7‒8.8)
High school graduate	10.5 (9.6‒11.5)	8.7 (7.9‒9.6)
More than high school	8.4 (8.1‒8.8)	6.4 (6.1‒6.7)
**Health insurance^¶^**		
Yes		
Any public	13.9 (13.1‒14.7)	11.6 (10.9‒12.4)
Any private	7.0 (6.7‒7.4)	5.2 (4.9‒5.5)
No insurance	6.3 (5.2‒7.7)	4.8 (3.9‒5.9)
Geographic classification**		
Urban/Suburban	8.5 (8.2‒9.0)	6.6 (6.2‒7.0)
Rural	10.5 (9.7‒11.4)	8.6 (7.9‒9.4)

**Abbreviations**: FPL = federal poverty level; NSCH = National Survey of Children’s Health.

* Estimates exclude other race and ethnicity groups that did not have a large enough sample size to produce stable estimates.

^†^ FPL is based on family income and family size using the Census Bureau’s poverty thresholds for the previous calendar year. Imputed income files were used to impute family income when it was not provided, and for NSCH family size was imputed using other information about the household when the number of family members was not provided (https://www.census.gov/topics/income-poverty/poverty/guidance/poverty-measures.html).

^§^ The highest level of parent education is based on the highest education level among up to two adults who were identified as primary caregivers in the survey.

^¶^ Private included any insurance from an employer or union, directly purchased, TRICARE or other military health care, or the Affordable Care Act; coverage from any government assistance plan was considered public, including Medicaid or other state-sponsored health plans including the Children’s Health Insurance Program. Respondents who indicated both public and private insurance coverage were represented in both subcategories.

** Method for determining geographic classification for NSCH was based on the 2010 Office of Management and Budget metropolitan and micropolitan statistical areas standards (https://www.govinfo.gov/content/pkg/FR-2010-06-28/pdf/2010-15605.pdf). Urban/suburban includes metropolitan statistical areas associated with at least one urbanized area of at least 50,000 population; rural was defined as counties that were not part of a metropolitan statistical area.

Children aged 6–11 years had higher prevalences of behavior problems than children who were aged <6 years or >11 years. Similar to the estimates of ADHD, boys had more than twice the estimated prevalence of behavior problems compared with girls. Black children had the highest estimated prevalence of behavior problems, followed by White and Hispanic children, with the lowest estimates among Asian children. Regarding socioeconomic factors, the highest prevalence of behavior problems was among children in homes affected by poverty and among children with public health insurance; the prevalence of behavior problems was also higher among children with parents who had a high school education (only) compared with those with more than a high school education. The prevalence of behavior problems was higher among children living in rural areas than among those in urban or suburban areas.

### Depression

Depressive disorder is characterized by significant feelings of sadness, hopelessness, or loss of interest or pleasure in daily activities, with symptoms persisting on most days for a 2-week period (*1*). Children and adolescents who have depression are at higher risk for other mental disorders and health conditions, as well as school problems, difficulty with social relationships, self-harm, and suicide (*17*,*34*). NSCH asked parents if a health care provider had ever told them that their child had depression and whether the child currently had the condition. NSDUH identified MDEs based on adolescent self-reports of depression symptoms, identifying ever having had an MDE and having had an MDE in the past year. For NHANES, adolescents reported on depression symptoms during the past 2 weeks. YRBS examined self-reported persistent feelings of sadness or hopelessness among high school students, primarily aged 14–18 years.

According to data from the 2016–2019 survey years of NSCH, an estimated 4.4% children and adolescents aged 3–17 years ever had diagnosed depression, and 3.4% had current depression (Table 6). Results from the 2018–2019 survey years of NSDUH indicated that 20.9% of adolescents aged 12–17 years ever had an MDE and 15.1% had an MDE in the past year. NHANES 2013–2018 data indicated that 5.8% of adolescents aged 12–17 years reported having major depression during the past 2 weeks, whereas YRBS 2019 data indicated that 36.7% of high school students aged primarily 14–18 years experienced persistent feelings of sadness or hopelessness during the past year. State prevalence estimates, based on NSCH data, of ever having been diagnosed with depression among children and adolescents aged 3–17 years ranged from 1.8% in Hawaii to 8.1% in Montana; estimates of current depression ranged from 1.4% in Hawaii to 6.6% in Montana (Supplementary Tables 1 and 2; https://stacks.cdc.gov/view/cdc/113924). On the basis of NSDUH data, the prevalence of ever having had an MDE among adolescents aged 12–17 years ranged from 13.1% in DC to 28.0% in Oregon. Similarly, the prevalence of past year MDEs among adolescents aged 12–17 years ranged from 9.8% in DC to 19.9% in New Mexico and Oregon (Supplementary Tables 1 and 2). YRBS state estimates are available online (https://yrbs-explorer.services.cdc.gov).

**TABLE 6:** Weighted prevalence estimates of surveillance indicators of depression among children and adolescents aged 3–17 years, by sociodemographic characteristics and year — four surveillance systems, United States, 2013–2019

Characteristic	Ever had depression (parent reported diagnosis)	Current depression (parent report)	Ever had major depressive episode (self-report)	Past year major depressive episode (self-report)	Current depression (self-report)*	Past year persistent feelings of sadness or hopelessness (self-report)^†^
	NSCH 2016–2019	NSCH 2016–2019	NSDUH 2018–2019	NSDUH 2018–2019	NHANES 2013–2018	YRBS 2019
	% (95% CI)	% (95% CI)	% (95% CI)	% (95% CI)	% (95% CI)	% (95% CI)
**Age group (yrs)**	3–17	3–17	12–17	12–17	12–17	~14–18^§^
**Sample size (no.)**	114,476	114,316	33,678	33,678	2,771	13,677
**Total**	**4.4 (4.2–4.7)**	**3.4 (3.2‒3.6)**	**20.9 (20.4‒21.6)**	**15.1 (14.6‒15.6)**	**5.8 (4.6‒7.4)**	**36.7 (35.1‒38.3)**
**Age group (yrs)**						
3–5	0.1 (0.1–0.3)	0.1 (0.1‒0.3)	NA	NA	NA	NA
6–11	2.3 (2.0–2.6)	1.9 (1.6‒2.2)	NA	NA	NA	NA
12–17	8.6 (8.1–9.1)	6.5 (6.1‒6.9)	20.9 (20.4‒21.6)	15.1 (14.6‒15.6)	5.8 (4.6‒7.4)	36.4 (34.8–38.0)
**Sex**						
Male	4.0 (3.7–4.4)	3.0 (2.8‒3.3)	11.8 (11.2‒12.5)	8.2 (7.7 ‒8.8)	3.3 (2.3‒4.6)	26.8 (25.2‒28.4)
Female	4.8 (4.5–5.2)	3.8 (3.5‒4.1)	30.4 (29.5‒31.4)	22.3 (21.4‒23.1)	8.4 (6.3‒11.1)	46.6 (44.4‒48.9)
**Race/Ethnicity** ^¶^						
Hispanic	4.0 (3.4–4.8)	2.7 (2.2‒3.2)	22.4 (21.1‒23.7)	16.2 (15.1‒17.4)	5.3 (3.7‒7.2)	40.0 (38.0‒42.1)
Black, non-Hispanic	4.5 (3.8–5.3)	3.7 (3.1‒4.4)	15.9 (14.6‒17.4)	10.8 (9.7‒12.0)	6.0 (4.1‒8.4)	31.5 (28.8‒34.3)
White, non-Hispanic	4.8 (4.6–5.1)	3.8 (3.6‒4.1)	21.4 (20.6‒22.2)	15.5 (14.8‒16.2)	6.0 (4.0‒8.6)	36.0 (34.1‒38.0)
Asian	1.6 (1.1–2.3)	1.3 (0.8‒2.0)	20.3 (17.4‒23.5)	14.3 (11.8‒17.2)	3.6 (1.5‒7.3)**	31.6 (27.4‒36.1)
American Indian or Alaska Native, non-Hispanic	NA	NA	18.2 (13.5–23.9)	13.6 (9.3–19.6)	NA	45.5 (32.7-58.9)
Native Hawaiian or other Pacific Islander, non-Hispanic	NA	NA	NA	NA	NA	36.8 (22.6‒53.7)
**FPL^††^**						
≤100% FPL	5.8 (5.1–6.6)	4.7 (4.1‒5.4)	18.6 (17.4‒19.8)	13.2 (12.3‒14.3)	8.5 (6.4‒11.0)	NA
>100% to ≤200% FPL	4.6 (4.1–5.2)	3.6 (3.2‒4.1)	21.8 (20.5‒23.1)	15.4 (14.3‒16.6)	6.3 (4.3‒8.9)	NA
>200% FPL	3.9 (3.6–4.2)	2.9 (2.7‒3.1)	21.5 (20.7‒22.3)	15.6 (15.0‒16.3)	4.9 (3.2‒7.2)	NA
**Highest level of parent education^§§^**						
Less than high school	5.3 (4.1–6.8)	3.8 (2.9‒5.0)	NA	NA	5.1 (3.0‒8.2)	NA
High school graduate	5.0 (4.4–5.7)	3.8 (3.4‒4.4)	NA	NA	12.4 (8.1‒18.0)	NA
More than high school	4.2 (3.9–4.4)	3.2 (3.0‒3.5)	NA	NA	4.4 (3.2‒7.0)	NA
**Health insurance^¶¶^**						
Yes						
Any public	6.3 (5.8–6.9)	5.0 (4.6‒5.5)	20.5 (19.5‒21.4)	14.5 (13.7 ‒15.3)	7.3 (5.6‒9.4)	NA
Any private	3.6 (3.4–3.9)	2.8 (2.6‒3.0)	21.3 (20.6‒22.2)	15.4 (14.7‒16.1)	4.8 (3.1‒7.0)	NA
No insurance	4.3 (3.1–6.0)	2.8 (2.0‒4.0)	20.2 (17.5‒23.2)	15.4 (13.1‒18.0)	3.8 (1.7‒7.0)	NA
Geographic classification***						
Urban/Suburban	4.3 (4.0–4.6)	3.2 (3.0‒3.5)	21.0 (20.3‒21.7)	15.2 (14.6‒15.8)	NA	36.6 (34.8‒38.5)
Rural	5.6 (5.0–6.4)	4.4 (3.8‒5.0)	20.7 (19.4‒22.1)	14.6 (13.5‒15.8)	NA	36.6 (33.5‒39.9)

**Abbreviations**: FPL = federal poverty level; NA = not available; NHANES = National Health and Nutrition Examination Survey; NSCH = National Survey of Children’s Health; NSDUH = National Survey on Drug Use and Health; YRBS = National Youth Risk Behavior Survey.

* Depression during past 2 weeks (score of ≥10 on the nine-item Patient Health Questionnaire depression module; adolescent report).

^†^ During the past 12 months before the survey, felt so sad or hopeless almost every day for 2 or more weeks in a row that they stopped doing some usual activities.

^§^ For YRBS, survey participants were public and private high school students in grades 9–12 (i.e., primarily aged 14–18 years).

^¶^ Estimates exclude other race and ethnicity groups that did not have a large enough sample size to produce stable estimates.

** Estimate did not meet all NCHS data presentation standards (CI width >5, relative CI width >130) and should be interpreted with caution.

^††^ For NSCH, NHIS, and NSDUH, FPL is based on family income and family size using the Census Bureau’s poverty thresholds for the previous calendar year. Imputed income files were used to impute family income when it was not provided, and for NSCH, family size was imputed using other information about the household when the number of family members was not provided (https://www.census.gov/topics/income-poverty/poverty/guidance/poverty-measures.html). NHANES uses the US Department of Health and Human Services poverty guidelines (https://aspe.hhs.gov/topics/poverty-economic-mobility/poverty-guidelines) to calculate FPLs (also known as the family income to poverty ratio) and does not impute missing incomes. NSDUH only imputes family size when exact counts cannot be determined from the household roster.

^§§^ The highest level of parent education is based on the highest education level among up to two adults who were identified as primary caregivers in the survey. For NHANES, education of household reference person and spouse of household reference person (most often primary caregiver of youth).

^¶¶^ Private included any insurance from an employer or union, directly purchased, TRICARE or other military health care, or the Affordable Care Act; coverage from any government assistance plan was considered public, including Medicaid or other state-sponsored health plans including the Children’s Health Insurance Program. Respondents who indicated both public and private insurance coverage were represented in both subcategories.

*** Methods for determining geographic classification differed by survey. *NSCH:* 2010 Office of Management and Budget metropolitan and micropolitan statistical areas standards (https://www.govinfo.gov/content/pkg/FR-2010-06-28/pdf/2010-15605.pdf). Urban/suburban includes metropolitan statistical areas associated with at least one urbanized area of at least 50,000 population; rural was defined as counties that were not part of a metropolitan statistical area; *NHIS:* 2013 NCHS urban/rural classification (https://www.cdc.gov/nchs/data/series/sr_02/sr02_166.pdf). Urban/suburban includes large metropolitan, medium metropolitan, and small metropolitan areas, whereas rural includes nonmetropolitan areas with >50,000 population; *NSDUH:* Rural-Urban Continuum Codes (https://www.ers.usda.gov/data-products/rural-urban-continuum-codes/). Urban/suburban includes large metropolitan, medium metropolitan, and small metropolitan areas; rural includes nonmetropolitan counties. *YRBS:* MDR (formerly Market Data Retrieval) propriety information, determined by an MDR formula based on the National Center for Education Statistics Locale Code classification and zip code. The urban category includes the urban, suburban, and town groups, and the rural category includes the rural/nonmetropolitan group.

Parent-reported data from NSCH during the 2016–2019 survey years indicated that the prevalence of ever having had a diagnosis of depression increased with age, particularly in adolescence; the prevalence was 0.1% for children aged 3–5 years, 2.3% for children aged 6–11 years, and 8.6% for adolescents aged 12–17 years. Among adolescents aged 12–17 years, parents reported a prevalence of 6.5% for current depression, which is slightly higher than the NHANES (2013–2018) estimate of 5.8% for adolescent self-reported current depression symptoms but lower than estimates of ever having had an MDE (20.9%) and past year MDE (15.1%) based on 2018–2019 NSDUH data. Across all measures of depression, girls had higher a prevalence than boys, with estimates derived from adolescent self-report (2018–2019 NSDUH, 2013–2018 NHANES, and 2019 YRBS) approximately twice as high for girls as boys. Among racial and ethnic groups, patterns differed depending on the survey. The lowest prevalence of diagnosed depression was among Asian children and adolescents aged 3–17 years according to NSCH data; otherwise, few differences regarding diagnosis were observed among Hispanic, White, Black, or AI/AN children and adolescents. Estimates of current depression among children and adolescents aged 3–17 years based on NSCH data were lower among Hispanic children and adolescents than among White children and adolescents; this difference was not observed with NHANES data for current depression among adolescents aged 12–17 years. Prevalence estimates of MDE among adolescents aged 12–17 years based on NSDUH data were lower among Black adolescents than among Hispanic and White adolescents. YRBS data indicated that among high school students primarily aged 14–18 years, the prevalence of persistent feelings of sadness or hopelessness was higher among Hispanic students than among Black, White, and Asian students. The estimated percentages of AI/AN and NH/OPI students primarily aged 14­–18 years with persistent feelings of sadness or hopelessness were similar to the estimates for the other racial and ethnic groups.

An association was found between household poverty level and prevalence of depression (NSCH 2016–2019 and NHANES 2013–2018), with the lowest prevalence of depression among children from households with the highest income level (>200% FPL). However, NSDUH estimates of ever and past year MDE were higher for children from households with the highest income level (>200% FPL) compared with those at the lowest income level (≤100% FPL). According to NSCH data, the prevalence of depression in children with any private health insurance or no health insurance was lower than the prevalence for children with any public health insurance; otherwise, estimates using NSDUH (2018–2019) and NHANES data on health insurance statuses did not differ. NSCH data indicated that children from rural areas had a higher prevalence of diagnosed depression than those from other areas. Depression indicators from the 2018­–2019 NSDUH and the 2019 YRBS data indicated no differences by geographic classification.

### Anxiety

Anxiety disorders are characterized by excessive fears and worries that are developmentally inappropriate, persist for >6 months, are severe, and interfere with daily functioning (*1*). Anxiety symptoms in childhood and adolescence might include clear fear or worry but can also include irritability, anger, and trouble sleeping, as well as physical symptoms such as fatigue, headaches, or stomachaches (*1*). In addition to generalized anxiety, anxiety can also manifest as separation anxiety, panic disorder, or phobias. Children with anxiety disorders are at risk for other mental disorders and physical health conditions, as well as school problems and negative effects on family relationships (*17*,*34*,*42*). Only NSCH asked parents whether a health care provider had ever told them that their child had anxiety problems, followed by a question on whether the child currently had anxiety.

Results of the 2016–2019 survey years of NSCH showed that 9.4% of children and adolescents aged 3–17 years had ever received a diagnosis of anxiety problems, and 7.8% had anxiety problems at the time of the survey (Table 7). State prevalence estimates of ever having had anxiety problems among children and adolescents aged 3–17 years ranged from 5.1% in Hawaii to 17.3% in Maine; estimates of current anxiety ranged from 3.8% in Hawaii to 14.7% in Maine (Supplementary Tables 1 and 2; https://stacks.cdc.gov/view/cdc/113924).

**TABLE 7:** Weighted prevalence estimates of anxiety problems among children and adolescents aged 3–17 years, by sociodemographic characteristics — National Survey of Children's Health, United States, 2016–2019

Characteristic	Ever had anxiety problems	Current anxiety problems
	% (95% CI)	% (95% CI)
**Age group (yrs)**	3–17	3–17
**Sample size (no.)**	114,476	114,476
**Total**	**9.4 (9.0‒9.7)**	**7.8 (7.5‒8.1)**
**Age group (yrs)**		
3–5	2.0 (1.7–2.4)	1.6 (1.4–2.0)
6–11	8.6 (8.1–9.2)	7.1 (6.6–7.6)
12–17	13.7 (13.1–14.3)	11.4 (10.9–12.0)
**Sex**		
Male	9.1 (8.6–9.6)	7.5 (7.1–7.9)
Female	9.7 (9.2–10.2)	8.1 (7.6–8.5)
Race/Ethnicity*		
Hispanic	8.0 (7.1‒9.0)	6.1 (5.3‒6.9)
Black, non-Hispanic	6.4 (5.6‒7.3)	5.3 (4.6‒6.1)
White, non-Hispanic	11.4 (11.0‒11.8)	9.7 (9.4‒10.1)
Asian, non-Hispanic	3.0 (2.3‒3.8)	2.2 (1.7‒2.9)
**FPL^†^**		
≤100% FPL	9.7 (8.8‒10.6)	8.0 (7.2‒8.8)
>100% to ≤ 200% FPL	9.4 (8.6‒10.3)	7.5 (6.9‒8.3)
>200% FPL	9.3 (8.9‒9.7)	7.8 (7.4‒8.2)
**Highest level of parent education^§^**		
Less than high school	8.9 (7.3‒10.8)	6.3 (5.1‒7.6)
High school graduate	9.1 (8.3‒10.0)	7.4 (6.7‒8.2)
More than high school	9.6 (9.2‒9.9)	8.1 (7.8‒8.5)
**Health insurance^¶^**		
Yes		
Any public	11.3 (10.6‒12.1)	9.4 (8.8‒10.0)
Any private	9.2 (8.8‒9.6)	7.6 (7.3‒8.0)
No insurance	6.9 (5.6‒8.6)	5.6 (4.5‒6.9)
Geographic classification**		
Urban/Suburban	9.0 (8.6‒9.5)	7.4 (7.1‒7.8)
Rural	10.2 (9.4‒11.1)	8.7 (7.9‒9.4)

**Abbreviations:** FPL = federal poverty level; NSCH = National Survey of Children’s Health.

* Estimates exclude other race and ethnicity groups that did not have a large enough sample size to produce stable estimates

^†^ FPL is based on family income and family size using the Census Bureau’s poverty thresholds for the previous calendar year. Imputed income files were used to impute family income when it was not provided and family size was imputed using other information about the household when the number of family members was not provided (https://www.census.gov/topics/income-poverty/poverty/guidance/poverty-measures.html)

^§^ The highest level of parent education is based on the highest education level among up to two adults who were identified as primary caregivers in the survey.

^¶^ Private included any insurance from an employer or union, directly purchased, TRICARE or other military health care, or the Affordable Care Act; coverage from any government assistance plan was considered public, including Medicaid or other state-sponsored health plans including the Children’s Health Insurance Program. Respondents who indicated both public and private insurance coverage were represented in both subcategories.

** Method for determining geographic classification for NSCH was based on the 2010 Office of Management and Budget metropolitan and micropolitan statistical areas standards (https://www.govinfo.gov/content/pkg/FR-2010-06-28/pdf/2010-15605.pdf). Urban/suburban includes metropolitan statistical areas associated with at least one urbanized area of at least 50,000 population; rural was defined as counties that were not part of a metropolitan statistical area.

The prevalence of ever having had anxiety problems increased with age, from 2.0% among children aged 3–5 years to 8.6% for those aged 6–11 years and 13.7% for those aged 12–17 years. Estimates of anxiety for boys and girls were similar. The highest prevalence estimates among children and adolescents aged 3–17 years were observed among White children and adolescents, and the lowest were observed among Asian children and adolescents. Although no differences in the prevalence of ever having anxiety problems were found by parent education level, prevalence estimates of current anxiety among children and adolescents aged 3–17 years were higher among children and adolescents of parents with more than a high school education than among those with less than a high school education. Prevalence estimates were highest for children and adolescents aged 3–17 years with public health insurance and lowest for those with no health insurance. No differences were found by poverty level. Prevalence estimates of anxiety among children and adolescents aged 3–17 years were slightly higher for children and adolescents living in rural areas than in urban areas.

### Autism Spectrum Disorder

ASD is a developmental disability that can cause significant social, communication, and behavioral challenges. Symptoms include difficulties with communication, social interactions, restricted and repetitive behaviors, and adapting to change. Intellectual ability can range from gifted to severely challenged (*1*). A diagnosis of ASD now encompasses several conditions that were previously diagnosed separately: autistic disorder, pervasive developmental disorder not otherwise specified, and Asperger syndrome. The DSM-5, published in 2013, collectively described these conditions as ASD (*1*). Children with ASD often experience significant functional impairment and are at risk for having other mental disorders and medical conditions, including respiratory, gastrointestinal, dermatologic, and neurologic conditions that require treatment (*81*). NSCH and NHIS surveys both asked parents if a health care provider had ever told them that their child had ASD, followed by a question asking whether the child currently had ASD. The ADDM Network uses a records review to cumulatively assess ASD diagnoses and symptoms through age 8 years.

Estimates of the overall prevalence of ASD among children and adolescents aged 3–17 years using 2016–2019 NSCH data were 3.1% and 2.9% for ever having had a diagnosis of ASD and having a current diagnosis of ASD, respectively (Table 8). Estimates of ASD among children and adolescents aged 3–17 years from 2017–2018 NHIS data were lower, at 2.4% and 2.0%, respectively. State prevalence estimates of ever ASD among children and adolescents aged 3–17 years ranged from 1.5% in North Dakota to 4.5% in Delaware. Estimates of current ASD ranged from 1.3% in North Dakota to 4.1% in Delaware (Supplementary Tables 1 and 2; https://stacks.cdc.gov/view/cdc/113924).

**TABLE 8:** Weighted prevalence estimates of autism spectrum disorder among children and adolescents aged 3–17 years, by sociodemographic characteristics — three surveillance systems, United States, 2016–2019

Characteristic	Ever had ASD		Current ASD		Met ASD surveillance case definition*
	NSCH 2016–2019	NHIS 2017–2018	NSCH 2016–2019	NHIS 2017–2018	ADDM Network 2016
	% (95% CI)	% (95% CI)	% (95% CI)	% (95% CI)	% (95% CI)
**Age group (yrs)**	3–17	3–17	3–17	3–17	8
**Sample size (no.)**	114,476	14,316	114,476	14,292	275,419
**Total**	**3.1 (2.8‒3.3)**	**2.4 (2.1‒2.7)**	**2.9 (2.6‒3.1)**	**2.0 (1.8‒2.4)**	**1.9 (1.8‒1.9)**
**Age group (yrs)**					
3–5	2.1 (1.7‒2.5)	1.8 (1.3‒2.6)	1.9 (1.6‒2.2)	1.6 (1.1‒2.3)	NA
6–11	3.2 (2.8‒3.6)	2.3 (1.8‒3.0)	3.0 (2.6‒3.4)	2.0 (1.5‒2.5)	1.9 (1.8‒1.9)^†^
12–17	3.5 (3.1‒3.9)	2.7 (2.2‒3.2)	3.3 (2.9‒3.7)	2.3 (1.9‒2.8)	NA
**Sex**					
Male	4.8 (4.3‒5.2)	3.6 (3.1‒4.2)	4.4 (4.0‒4.9)	3.1 (2.7‒3.7)	3.0 (2.9‒3.1)
Female	1.3 (1.1‒1.5)	1.1 (0.8‒1.5)	1.2 (1.0‒1.5)	0.9 (0.7‒1.3)	0.7 (0.7‒0.7)
**Race/Ethnicity^§^**					
Hispanic	3.5 (2.8‒4.4)	2.1 (1.6‒2.7)	3.4 (2.7‒4.3)	1.8 (1.3‒2.4)	1.5 (1.4‒1.6)
Black, non-Hispanic	3.4 (2.7‒4.1)	2.8 (1.7‒4.6)	3.1 (2.6‒3.8)	2.2 (1.4‒3.4)	1.8 (1.7‒1.9)
White, non-Hispanic	2.9 (2.7‒3.1)	2.5 (2.1‒2.9)	2.7 (2.5‒2.9)	2.3 (1.9‒2.7)	1.9 (1.8‒1.9)
Asian, non-Hispanic	2.1 (1.6‒2.8)	1.3 (0.7‒2.5)	1.9 (1.4‒2.6)	0.8 (0.4‒1.7)	1.8 (1.6‒2.0)
**FPL^¶^**					
≤100% FPL	4.0 (3.3‒4.9)	2.3 (1.6‒3.1)	3.9 (3.2‒4.8)	2.1 (1.5‒3.0)	NA
>100% to ≤200% FPL	3.8 (3.2‒4.6)	2.8 (2.2‒3.5)	3.5 (2.9‒4.3)	2.3 (1.8‒3.0)	NA
>200% FPL	2.4 (2.3‒2.6)	2.3 (1.8‒2.8)	2.3 (2.1‒2.5)	1.9 (1.6‒2.3)	NA
Highest level of parent education**					
Less than high school	3.1 (2.1‒4.8)	1.6 (1.0‒2.6)	3.1 (2.0‒4.7)	1.4 (0.8‒2.3)	NA
High school graduate	3.6 (3.0‒4.4)	2.2 (1.6‒2.9)	3.4 (2.7‒4.2)	2.0 (1.5‒2.8)	NA
More than high school	2.9 (2.7‒3.2)	2.5 (2.1‒3.0)	2.7 (2.5‒2.9)	2.1 (1.8‒2.5)	NA
**Health insurance^††^**					
Yes					
Any public	4.8 (4.3‒5.5)	3.1 (2.6‒3.7)	4.6 (4.0‒5.2)	2.8 (2.3‒3.4)	NA
Any private	2.6 (2.4‒2.9)	2.2 (1.8‒2.8)	2.4 (2.2‒2.7)	1.8 (1.4‒2.3)	NA
No insurance	1.8 (1.1‒3.0)	2.4 (1.0‒5.3)	1.8 (1.1‒3.0)	1.7 (0.7‒4.2)	NA
**Geographic classification^§§^**					
Urban/Suburban	3.0 (2.7‒3.4)	2.3 (2.0‒2.7)	2.9 (2.6‒3.2)	2.0 (1.7‒2.3)	NA
Rural	2.8 (2.4‒3.2)	2.8 (2.0‒3.9)	2.6 (2.2‒3.0)	2.7 (1.9‒3.8)	NA

**Abbreviations**: ADDM = Autism and Developmental Disabilities Monitoring; ASD = autism spectrum disorder; FPL = federal poverty level; NA = not available; NHIS = National Health Interview Survey; NSCH = National Survey of Children’s Health.

* Case definition based on *Diagnostic and Statistical Manual of Mental Disorders*, *5th Edition* criteria for autism spectrum disorder. Clinicians applied the case definition through a review of information systematically collected from developmental evaluations completed by medical and educational service providers in the community.

^†^ Estimate is for children aged 8 years only.

^§^ Estimates exclude other race and ethnicity groups that did not have a large enough sample size to produce stable estimates

^¶^ FPL is based on family income and family size using the Census Bureau’s poverty thresholds for the previous calendar year. Imputed income files were used to impute family income when it was not provided, and for NSCH, family size was imputed using other information about the household when the number of family members was not provided (https://www.census.gov/topics/income-poverty/poverty/guidance/poverty-measures.html)

** The highest level of parent education is based on the highest education level among up to two adults who were identified as primary caregivers in the survey.

^††^ Private included any insurance from an employer or union, directly purchased, TRICARE or other military health care, or the Affordable Care Act; coverage from any government assistance plan was considered public, including Medicaid or other state-sponsored health plans including the Children’s Health Insurance Program. Respondents who indicated both public and private insurance coverage were represented in both subcategories.

^§§^ Method for determining geographic classification differed by survey. *NSCH:* 2010 Office of Management and Budget metropolitan and micropolitan statistical areas standards (https://www.govinfo.gov/content/pkg/FR-2010-06-28/pdf/2010-15605.pdf). Urban/suburban includes metropolitan statistical areas associated with at least one urbanized area of at least 50,000 population; rural was defined as counties that were not part of a metropolitan statistical area; *NHIS:* 2013 NCHS urban/rural classification (https://www.cdc.gov/nchs/data/series/sr_02/sr02_166.pdf). Urban/suburban includes large metropolitan, medium metropolitan, and small metropolitan areas, whereas rural includes nonmetropolitan areas with >50,000 population.

Prevalence estimates of ASD from 2016–2019 NSCH data increased from ages 3–5 years to ≥6 years. Although differences showed similar patterns in 2017–2018 NHIS data (a survey with a smaller sample size), the differences were not significant. The 1.9% estimate from 2016 ADDM Network data among children aged 8 years was lower than the 3.0% estimate of current ASD from NSCH data among children aged 6–11 years but similar to the 2.0% estimate from NHIS data among children aged 6–11 years. Across all data sources, ASD was more common in boys. Prevalence estimates among different racial and ethnic groups varied among the surveys. Whereas the ADDM data showed that Hispanic children aged 8 years had a lower prevalence of ASD than White or Black children aged 8 years, estimates of current ASD among children and adolescents aged 3–17 years based on NSCH data were lower among Asian children compared with Hispanic children. Other ASD estimates for children and adolescents aged 3–17 years from NSCH and NHIS data showed no differences among racial and ethnic groups. Regarding socioeconomic factors, NSCH data showed that children with public health insurance had the highest prevalence of ASD, and children and adolescents aged 3–17 years in families with the highest income level (>200% FPL) had the lowest prevalence of ASD; these differences were not observed in NHIS data. Parent education level and geographic classification were not associated with significantly different prevalences of ASD in NSCH or NHIS data.

### Tourette Syndrome

Tourette syndrome is a tic disorder characterized by the presence of both multiple motor tics and at least one vocal tic, with tics occurring for at least 1 year to meet diagnostic criteria. Other tic disorders include persistent motor tic disorder, with motor tics present for at least 1 year, and persistent vocal tic disorder, with vocal tics present for at least 1 year. Provisional tic disorder is used when any tics have been present for <1 year (*1*). Tics often begin in children aged 4–8 years, and approximately 80% have another co-occurring mental disorder, including ADHD, obsessive-compulsive disorder, anxiety, or depression (*1*,*82*). Tourette syndrome is the only tic disorder included in national surveillance systems and is only included in the NSCH questionnaire, which asks parents whether a health care provider has ever told them that their child had Tourette syndrome, followed by a question on whether the child currently has Tourette syndrome.

Data from the 2016–2019 survey years of NSCH indicate that 0.3% of children and adolescents aged 3–17 had ever received a diagnosis of Tourette syndrome, and 0.2% of children and adolescents aged 3–17 years had a current Tourette syndrome diagnosis (Table 9). A Tourette syndrome diagnosis was more common in boys than girls; prevalence estimates were similar among various age groups, races/ethnicities (Hispanic and White), parent education levels, types of health insurance, household poverty level, and geographic classification. Because of the relatively small sample size of children and adolescents aged 3–17 years with Tourette syndrome, state prevalence estimates are not presented, and estimates for certain sociodemographic characteristics were suppressed because of cell sizes of <20 (children aged 3–5 years, Black children, Asian children, children of parents with less than a high school education, and children with no health insurance).

**TABLE 9:** Weighted prevalence estimates of Tourette syndrome among children and adolescents aged 3–17 years, by sociodemographic characteristics — National Survey of Children's Health, United States, 2016–2019

Characteristic	Ever had Tourette syndrome	Current Tourette syndrome
	% (95% CI)	% (95% CI)
**Age group (yrs)**	3–17	3–17
**Sample size (no.)**	114,476	114,476
**Total**	**0.3 (0.2‒0.4)**	**0.2 (0.2‒0.3)**
**Characteristic**		
**Age group (yrs)**		
3–5	—*	—*
6–11	0.3 (0.2‒0.5)	0.2 (0.1‒0.4)
12–17	0.4 (0.3‒0.5)	0.3 (0.2‒0.4)
**Sex**		
Male	0.5 (0.3‒0.6)	0.4 (0.3‒0.5)
Female	0.1 (0.1‒0.2)	0.1 (0.0‒0.1)
**Race/Ethnicity^†^**		
Hispanic	0.3 (0.1‒0.6)	0.2 (0.1‒0.6)
Black, non-Hispanic	—*	—*
White, non-Hispanic	0.3 (0.3‒0.4)	0.3 (0.2‒0.4)
Asian, non-Hispanic	—*	—*
**FPL^§^**		
≤100% FPL	0.2 (0.2‒0.4)	0.2 (0.1‒0.3)
>100% to ≤200% FPL	0.2 (0.1‒0.3)	0.2 (0.1‒0.3)
>200% FPL	0.3 (0.2‒0.4)	0.2 (0.2‒0.4)
**Highest level of parent education^¶^**		
Less than high school	—*	—*
High school graduate	0.3 (0.2‒0.4)	0.2 (0.1‒0.3)
More than high school	0.3 (0.2‒0.4)	0.2 (0.2‒0.3)
Health insurance**		
Yes		
Any public	0.2 (0.2‒0.3)	0.2 (0.1‒0.3)
Any private	0.3 (0.2‒0.4)	0.2 (0.2‒0.4)
No insurance	0.4 (0.2‒0.8)	—*
**Geographic classification^††^**		
Urban/Suburban	0.3 (0.2‒0.4)	0.2 (0.2‒0.3)
Rural	0.4 (0.3‒0.6)	0.3 (0.2‒0.5)

**Abbreviations**: FPL = federal poverty level; NSCH = National Survey of Children’s Health.

* Estimates based on cell sizes <20 have been suppressed due to instability of estimates.

^†^ Estimates exclude other race and ethnicity groups that did not have a large enough sample size to produce stable estimates.

^§^ FPL is based on family income and family size using the Census Bureau’s poverty thresholds for the previous calendar year. Imputed income files were used to impute family income when it was not provided and family size was imputed using other information about the household when the number of family members was not provided (https://www.census.gov/topics/income-poverty/poverty/guidance/poverty-measures.html).

^¶^ The highest level of parent education is based on the highest education level among up to two adults who were identified as primary caregivers in the survey.

** Private included any insurance from an employer or union, directly purchased, TRICARE or other military health care, or the Affordable Care Act; coverage from any government assistance plan was considered public, including Medicaid or other state-sponsored health plans including the Children’s Health Insurance Program. Respondents who indicated both public and private insurance coverage were represented in both subcategories.

^††^ Method for determining geographic classification for NSCH was based on the 2010 Office of Management and Budget metropolitan and micropolitan statistical areas standards (https://www.govinfo.gov/content/pkg/FR-2010-06-28/pdf/2010-15605.pdf). Urban/suburban includes metropolitan statistical areas associated with at least one urbanized area of at least 50,000 population; rural was defined as counties that were not part of a metropolitan statistical area.

### Substance Use Disorders

Substance use is typically initiated during adolescence (*83*). Familial, social, and individual risk factors might lead to substance use and substance use disorders among youths (*84*). The use of substances during adolescence has been linked to motor vehicle deaths, sexually transmitted infections, and other physical and mental health problems (*84*). Substance use disorders are characterized by impairments caused by the recurrent use of alcohol or illicit drugs (or both), including health issues, disabilities, and failure to meet responsibilities at work, school, or home (*85*). Substance use disorders during the past year were assessed by NSDUH through adolescent self-report on use of and experiences related to use of alcohol and illicit drugs. During 2018–2019, an estimated 1 million adolescents aged 12–17 years met criteria for a past year substance use disorder (*85*) (Table 3). 

Data from the 2018 and 2019 NSDUH indicated that an estimated 407,000 adolescents aged 12–17 years (1.6%) had an alcohol use disorder. Alcohol use disorder among adolescents aged 12–17 years varied by state, with prevalence estimates ranging from 0.9% in Delaware to 4.7% in Vermont. Approximately 788,000 (3.2%) adolescents aged 12–17 years had an illicit drug use disorder. State prevalence estimates of an illicit drug disorder among adolescents aged 12–17 years ranged from 1.1% in Louisiana to 6.0% in Vermont. More detailed information on substance use disorders by state is available (Supplementary Table 2; https://stacks.cdc.gov/view/cdc/113924).

Past year alcohol use was higher among adolescent girls aged 12–17 years (2.0%) than among adolescent boys aged 12–17 years (1.3%) (Table 10). Overall substance use disorder (either alcohol or illicit drugs) was lower among Asian adolescents aged 12–17 years (2.0%) than among White (4.2%) and Hispanic (4.5%) adolescents aged 12–17 years. Alcohol use disorder was lower among Asian and Black (both 0.5%) adolescents aged 12–17 years than among Hispanic (1.7%) and White (2.0%) adolescents aged 12–17 years; estimates among Asian and Hispanic adolescents did not differ. A higher prevalence of illicit drug use disorder was reported by adolescents aged 12–17 years with public health insurance (3.6%) compared with those with private health insurance (2.8%). The prevalence of substance use disorders was similar across poverty levels and geographic classifications.

**TABLE 10:** Weighted prevalence estimates of past year substance use disorder, alcohol use disorder, and/or illicit drug use disorder among adolescents aged 12–17 years, by sociodemographic characteristics — National Survey on Drug Use and Health, United States, 2018–2019

Characteristic	Past year substance use disorder*	Past year alcohol use disorder	Past year illicit drug use disorder
	% (95% CI)	% (95% CI)	% (95% CI)
**Age group (yrs)**	12–17	12–17	12–17
**Sample size (no.)**	33,678	33,678	33,678
**Total**	**4.1 (3.8–4.4)**	**1.6 (1.5‒1.8)**	**3.2 (2.9‒3.4)**
**Characteristic**			
**Sex**			
Male	3.8 (3.4–4.2)	1.3 (1.1‒1.6)	3.1 (2.7‒3.4)
Female	4.4 (4.0–4.8)	2.0 (1.7‒2.2)	3.3 (2.9‒3.6)
**Race/Ethnicity^†^**			
Hispanic	4.5 (3.8–5.2)	1.7 (1.3‒2.2)	3.5 (2.9‒4.2)
Black, non-Hispanic	3.3 (2.7–4.0)	0.5 (0.3‒0.7)	3.0 (2.4‒3.7)
White, non-Hispanic	4.2 (3.9–4.6)	2.0 (1.8‒2.3)	3.1 (2.8‒3.4)
Asian, non-Hispanic	2.0 (1.1–3.4)	0.5 (0.2‒1.6)	1.7 (1.0‒3.0)
**FPL^§^**			
≤100% FPL	3.9 (3.4–4.6)	1.4 (1.1‒1.8)	3.2 (2.7‒3.8)
>100% to ≤200% FPL	4.3 (3.8–5.0)	1.5 (1.2‒1.9)	3.5 (3.0‒4.1)
>200% FPL	4.0 (3.7–4.4)	1.8 (1.5‒2.0)	3.0 (2.7‒3.4)
**Health insurance^¶^**			
Yes			
Any public	4.3 (3.9–4.8)	1.5 (1.2‒1.8)	3.6 (3.2‒4.1)
Any private	3.9 (3.5–4.3)	1.7 (1.5‒2.0)	2.8 (2.5‒3.1)
No insurance	4.8 (3.6–6.4)	1.6 (1.0‒2.4)	4.1 (3.0‒5.6)
Geographic classification**			
Urban/Suburban	4.1 (3.8–4.5)	1.6 (1.4‒1.8)	3.3 (3.0‒3.6)
Rural	3.8 (3.3–4.4)	2.1 (1.7‒2.5)	2.5 (2.1‒3.0)

**Abbreviations**: FPL = federal poverty level; NSDUH = National Survey on Drug Use and Health.

* Includes either past year alcohol use disorder or past year illicit drug use disorder.

^†^ Estimates exclude other race and ethnicity groups that did not have a large enough sample size to produce stable estimates.

^§^ FPL is based on family income and family size using the Census Bureau’s poverty thresholds for the previous calendar year. Imputed income files were used to impute family income when it was not provided (https://www.census.gov/topics/income-poverty/poverty/guidance/poverty-measures.html). NSDUH only imputes family size when exact counts cannot be determined from the household roster.

^¶^ Private included any insurance from an employer or union, directly purchased, TRICARE or other military health care, or the Affordable Care Act; coverage from any government assistance plan was considered public, including Medicaid or other state-sponsored health plans including the Children’s Health Insurance Program. Respondents who indicated both public and private insurance coverage were represented in both subcategories.

** Geographic classification for NSDUH used Rural-Urban Continuum Codes (https://www.ers.usda.gov/data-products/rural-urban-continuum-codes/). Urban/suburban includes large metropolitan, medium metropolitan, and small metropolitan areas; rural includes nonmetropolitan counties.

### Suicide

Suicide is defined as a death caused by injuring oneself with the intent to die. Suicidal behavior is a public health problem (*86*) that can have lasting effects on persons, families, and communities associated with the decedent (*87*). Suicide is usually the result of a combination of individual, relational, community, and societal factors that interact with one another over time (*87*). For example, suicide is associated with violence victimization, such as child abuse and neglect, bullying, peer violence, and dating and sexual violence (*88*). Suicide does not always result from a mental disorder (*78*). A study of circumstances surrounding suicides documented in NVDRS found that 54% of suicide decedents did not have a known mental health problem (*86*). The number of deaths from suicides reflects only a small portion of the overall impact of suicidal behavior. Although suicide is a complex problem, it can be prevented by using a public health approach that reduces factors that increase suicide risk and increases factors that promote resilience (*89*).

Data on suicide among children and adolescents were available from NVSS 2018–2019, NVDRS 2014–2018, and SAVD-SS 2013–2019; data on suicidal ideation were available from YRBS 2019. Data from YRBS indicated that during the past year among U.S. high school students primarily aged 14–18 years, 18.8% seriously considered attempting suicide, 15.7% made a suicide plan, 8.9% attempted suicide one or more times, and 2.5% made a suicide attempt requiring medical treatment (Table 11). On the basis of data from NVSS, with coverage of the entire United States in 2018 and 2019, a total of 5,744 (6.9 per 100,000) persons aged 10–19 years died by suicide; the corresponding number from NVDRS, with data from selected states and counties for 2014–2018, was 4,903 (7.0 per 100,000) suicides among persons aged 10–19 years. During January 2013–June 2019, SAVD-SS data included 85 suicides; of these, 47 (55.3%) occurred among persons aged 10–18 years, and the remaining 38 suicides (44.7%) involved persons aged ≥19 years. The number of school-associated suicides among persons aged 10–18 years fluctuated by year, ranging from four in the first 6 months of 2019 to 11 in 2013. Seven (14.9%) of these suicides involved a known diagnosed or suspected mental health condition. NVSS data indicate that rates of suicide per 100,000 persons aged 10–19 years were the lowest in Massachusetts (2.8) and the highest in Alaska (27.3) (Supplementary Table 2; https://stacks.cdc.gov/view/cdc/113924).

**TABLE 11:** Prevalence estimates* of suicidal ideation and suicide attempts and number and rate of suicides among persons aged 10–19 years, by sociodemographic characteristics and known circumstances — three surveillance systems, 2014‒2019

Characteristic	Seriously considered attempting suicide^†^	Made suicide plan^†^	Attempted suicide^§^	Made suicide attempt requiring medical treatment^¶^	Suicide				Current mental disorder among those who died by suicide	Current mental disorder treatment among those who died by suicide
	YRBS 2019 (N = 13,677)				NVSS 2018–2019**		NVDRS 2014–2018^††^		NVDRS 2014–2018^††^	
	% (95% CI)	% (95% CI)	% (95% CI)	% (95% CI)	No.	Rate per 100,000 (95% CI)	No.	Rate per 100,000 (95% CI)	No. (%)^§§^	No. (%)^§§^
**Age group (yrs)**	14–18^¶¶^	14–18^¶¶^	14–18^¶¶^	14–18^¶¶^	10–19	10–19	10–19	10–19	10–19	10–19
**Total**	**18.8 (17.6–20.0)**	**15.7 (14.6–16.9)**	**8.9 (7.9–10.0)**	**2.5 (2.1–3.0)**	**5,744**	**6.9 (6.7‒7.0)**	**4,903**	**7.0 (4.7‒9.3)**	**2,100 (46.9)**	**1,325 (29.6)**
Age group (yrs)***										
10‒14	18.1 (15.1‒21.5)	14.8 (12.4‒17.6)	8.4 (7.0‒10.0)	1.9 (1.0‒3.4)	1,130	2.7 (2.6‒2.9)	928	2.7 (1.7‒3.7)	359 (8.0)	242 (5.4)
15‒19	18.8 (17.7‒19.9)	15.7 (14.7‒16.8)	8.9 (7.8‒10.1)	2.6 (2.1‒3.1)	4,614	10.9 (10.6‒11.3)	3,975	11.2 (9.1‒13.2)	1,741 (38.9)	1,083 (24.2)
**Sex**										
Male	13.3 (12.2‒14.5)	11.3 (10.3‒12.4)	6.6 (5.5‒8.1)	1.7 (1.3‒2.3)	4,286	10.0 (9.7‒10.3)	3,633	10.1 (8.2‒12.1)	1,404 (31.4)	848 (19.0)
Female	24.1 (22.3‒26.0)	19.9 (18.4‒21.6)	11.0 (9.7‒12.5)	3.3 (2.6‒4.2)	1,458	3.6 (3.4‒3.7)	1,270	3.7 (2.4‒4.9)	696 (15.6)	477 (10.7)
**Race/Ethnicity^†††^**										
Hispanic	17.2 (15.2‒19.4)	14.7 (13.0‒16.7)	8.9 (7.1‒11.1)	3.0 (2.3‒3.8)	978	4.7 (4.4‒5.0)	555	5.5 (4.7‒6.3)	203 (4.5)	111 (2.5)
Black, non-Hispanic	16.9 (15.3‒18.7)	15.0 (12.9‒17.5)	11.8 (8.7‒15.9)	3.3 (2.2‒4.9)	632	5.0 (4.6‒5.4)	543	4.3 (3.6‒5.1)	161 (3.6)	99 (2.2)
White, non-Hispanic	19.1 (17.6‒20.8)	15.7 (14.1‒17.4)	7.9 (6.9‒9.1)	2.1 (1.5‒2.8)	3,652	8.1 (7.9‒8.4)	3,395	7.8 (5.9‒9.7)	1,591 (35.6)	1,020 (22.8)
Asian, non-Hispanic	19.7 (15.8‒24.3)	16.1 (13.1‒19.7)	7.7 (4.8‒12.3)	1.7 (0.6‒4.6)	272	5.5 (4.8‒6.1)	144	4.5 (4.1‒4.9)	45 (1.0)	32 (0.72)
American Indian or Alaska Native, non-Hispanic	34.7 (23.8‒47.6)	24.2 (13.5‒39.6)	25.5 (12.6‒44.6)	11.5 (3.7‒30.3)	198	24.0 (20.7‒27.4)	NA	13.4 (13.1‒13.8)	NA	NA
Native Hawaiian or other Pacific Islander, non-Hispanic	15.4 (8.2‒27.0)	13.5 (6.2‒26.8)	8.8 (2.4‒27.2)	NA	NA	NA	NA	NA	NA	NA
**Geographic classification^§§§^**										
Urban/Suburban	19.0 (17.6‒20.5)	15.8 (14.5‒17.1)	8.9 (7.7‒10.2)	2.5 (2.0‒3.0)	4,559	6.3 (6.1‒6.5)	NA	NA	NA	NA
Rural	17.6 (16.0‒19.3)	15.0 (13.6‒16.6)	9.1 (7.3‒11.3)	2.8 (1.9‒4.1)	1,185	10.2 (9.6‒10.7)	NA	NA	NA	NA

**Abbreviations**: FPL = federal poverty level; NA = not available; NVDRS = National Violent Death Reporting System; NVSS = National Vital Statistics System; YRBS = National Youth Risk Behavior Survey.

* Estimates for YRBS are weighted; numbers, rates, and unweighted percentages are presented for NVSS and NVDRS.

^†^ During the 12 months before the survey.

^§^ During the 12 months before the survey, actually attempted suicide ≥1 time.

^¶^ During the 12 months before the survey, made a suicide attempt ≥1 time that resulted in injury, poisoning, or overdose that had to be treated by a physician or nurse.

** Suicides are identified using *International Classification of Diseases, 10th Revision* underlying cause-of-death codes U03, X60–X84, and Y87.0.

^††^ States (n = 18) included Alaska, Colorado, Georgia, Kentucky, Maryland, Massachusetts, Michigan, New Jersey, New Mexico, North Carolina, Ohio, Oklahoma, Oregon, Rhode Island, South Carolina, Utah, Virginia, and Wisconsin.

^§§^ The overall denominator for percent of suicides associated with a current mental disorder and current mental disorder treatment is 4,471.

^¶¶^ Survey participants were public and private high school students in grades 9–12 (i.e., primarily aged 14–18 years).

*** For YRBS, only age 14 years is included for the 10–14 years age group, and 15 to ≥18 years is included in 15–19 years age group.

^†††^ Estimates exclude other race and ethnicity groups that did not have a large enough sample size to produce stable estimates.

^§§§^ Geographic classification for YRBS was determined using MDR (formerly Market Data Retrieval) propriety information, determined by an MDR formula based on the National Center for Education Statistics Locale Code classification and zip code. The urban category includes the urban, suburban, and town groups, and the rural category includes the rural/nonmetropolitan group. Geographic classification for NVSS was determined by the decedent’s county of residence and was categorized using the 2013 NCHS Urban–Rural Classification Scheme for Counties. Counties were classified into six urbanization levels based on metropolitan–nonmetropolitan status, population distribution, and other factors. The four metropolitan categories (i.e., large central metro, large fringe metro, medium metro, and small metro) were grouped as urban counties. The two nonmetropolitan categories (i.e., micropolitan and noncore) were grouped as rural counties.

YRBS 2019 data indicate that among high school students primarily aged 14–18 years, the prevalence of suicidal ideation (i.e., seriously considered attempting suicide and made a suicide plan), attempting suicide, and making a suicide attempt requiring medical treatment were more common among female than male high school students. The prevalence of seriously considering attempting suicide was higher among AI/AN students primarily aged 14–18 years compared with Hispanic, Black, and White students. Similarly, the estimated percentage of students who attempted suicide was higher among AI/AN students than among Hispanic, White, and Asian students, and the estimated percentage of students who made a suicide attempt requiring medical treatment was also higher among AI/AN students than White students, but not significantly different compared with Hispanic, Black, and Asian students. Both NVSS 2018–2019 and NVDRS 2014–2018 data indicated that suicide rate was higher among persons aged 15–19 years than among those aged 10–14 years and higher among boys than among girls, and the rate per 100,000 children and adolescents aged 10–19 years was highest for AI/AN students. The rate was also higher among White children and adolescents than among those in other racial and ethnic groups; however, the rates among White and Hispanic children based on NVDRS data did not differ.

### Mental Health Services

Mental health services include the assessment, diagnosis, and treatment of mental disorders. Options to treat mental disorders among children include psychological treatments as well as medication (*75*,*90*–*92*). Effective treatments can vary by age of the child, type of diagnosis and co-occurring conditions, and other circumstances. Psychological treatments are typically delivered by mental health professionals, including psychologists, psychiatrists, or counselors, but also might involve training parents and teachers to deliver interventions to children (*75*,*93*). Evidence-based psychological treatment can focus on the parent, child, or family and can be delivered individually or in groups (*94*). Examples of effective treatment approaches include parent training in behavior management, which has been documented as effective for ADHD and disruptive behavior disorders, particularly for younger children (*94*,*95*); applied behavior analysis for ASD (*93*); cognitive-behavioral therapy for disruptive behavior disorders, depression, and anxiety (*96*–*98*); interpersonal psychotherapy for adolescents with depression (*99*); or behavior therapy targeted to specific skills, such as cognitive-behavioral intervention for tics (*100*) or organizational training for children with ADHD (*101*), among others. However, the treatment that children receive is often different from the originally developed and tested programs with proven effectiveness, and providers might vary in their use of strategies and degree of fidelity with evidence-based approaches (*102*).

Medications might be prescribed by primary care providers and other licensed mental health providers (*103*). The Food and Drug Administration (FDA) has approved medications for childhood mental disorders such as ADHD, depression, anxiety, and ASD; however, many medications for mental disorders are prescribed without specific FDA approval for use in children and without following standard care guidelines (*104*,*105*). Medication might be more effective if carefully titrated and used in combination with behavioral treatment (*95*). Indicators of mental health services were included in NHIS, NSCH, NHANES, and NSDUH. NHIS and NSCH both included questions about receiving services from a mental health professional during the past 12 months. In addition, NHIS included a question about seeing a general physician because of an emotional or behavioral problem. Both NSCH and NHANES included questions about medication; NSCH asked about whether the child had taken any medication because of difficulties with emotions, concentration, or behavior during the past 12 months, whereas NHANES asked about use of psychotropic medication during the past 30 days. NSDUH included questions about receipt of specialty and nonspecialty mental health services. All estimates of mental health services were calculated for the population of children and were not restricted to children with diagnosed mental disorders.

Regarding treatment, data from the 2016–2019 survey years of NSCH indicated that 10.1% of children and adolescents aged 3–17 years, regardless of diagnoses, received any treatment or counseling from a mental health professional during the past 12 months (Table 12). Data from the 2017–2018 survey years of NHIS indicated that 9.6% of parents of children and adolescents aged 3–17 years had consulted with a mental health professional about the child’s health during the past 12 months, and 5.2% had consulted with a general physician about an emotional or behavioral problem of their child. Data from the 2013–2018 NHANES indicated that 9.8% of parents of children aged 4–17 years had consulted with a mental health professional during the past 12 months. Data from the 2018–2019 survey years of NSDUH indicate that 25.9% of adolescents aged 12–17 years reported receiving mental health services in the past year. 

**TABLE 12:** Weighted prevalence estimates of receipt of mental health treatment, services, and medication among children and adolescents aged 3‒17 years, by sociodemographic characteristics — four surveillance systems, United States, 2013–2019

Characteristic	Mental health treatment, professional*	Mental health consultation, professional^†^		Mental health consultation, general physician^§^	Mental health services^¶^	Past year medication for mental health problems**	Current medication for mental health problems^††^
	NSCH 2016–2019	NHIS 2017–2018	NHANES 2013–2018	NHIS 2017–2018	NSDUH 2018–2019	NSCH 2016–2019	NHANES 2013–2018
	% (95% CI)	% (95% CI)	% (95% CI)	% (95% CI)	% (95% CI)	% (95% CI)	% (95% CI)
**Age group (yrs)**	3–17	3–17	4–17	3–17	12‒17	3–17	3–17
**Sample size (no.)**	114,476	14,287	8,071	13,440	33,678	114,476	8,637
**Total**	**10.1 (9.8‒10.5)**	**9.6 (9.0‒10.2)**	**9.8 (8.6‒11.2)**	**5.2 (4.8‒5.7)**	**25.9 (25.3–26.5)**	**7.8 (7.5‒8.1)**	**6.6 (5.7‒7.7)**
**Characteristic**							
**Age group (yrs)**							
3–5	2.6 (2.2‒3.1)	4.0 (3.1‒5.2)	4.7 (3.2‒6.5)	3.8 (3.0‒4.9)	NA	1.0 (0.7‒1.4)	1.2 (0.6‒2.0)
6–11	9.5 (9.0‒10.0)	9.5 (8.6‒10.5)	9.8 (8.3‒11.4)	5.5 (4.9‒6.3)	NA	7.2 (6.7‒7.6)	7.1 (5.9‒8.5)
12–17	14.3 (13.7‒15.0)	12.4 (11.5‒13.4)	11.5 (9.8‒13.5)	5.6 (5.0‒6.3)	25.9 (25.3–26.5)	11.6 (11.1‒12.2)	8.7 (7.0‒10.7)
**Sex**							
Male	10.6 (10.1‒11.1)	10.6 (9.8‒11.5)	10.9 (9.5‒12.5)	6.0 (5.3‒6.7)	21.3 (20.5–22.1)	9.5 (9.0‒9.9)	8.5 (7.3‒9.8)
Female	9.6 (9.1‒10.1)	8.6 (7.8‒9.4)	8.7 (7.3‒10.3)	4.5 (3.9‒5.0)	30.6 (29.7–31.5)	6.0 (5.6‒6.4)	4.7 (3.6‒6.0)
**Race/Ethnicity^§§^**							
Hispanic	8.7 (7.8‒9.6)	6.7 (5.8‒7.8)	7.4 (5.6‒9.6)	4.2 (3.5‒5.1)	24.6 (23.3–26.0)	5.3 (4.6‒6.0)	2.9 (1.9‒4.1)
Black, non-Hispanic	9.8 (8.8‒10.9)	7.6 (6.2‒9.2)	8.8 (7.4‒10.4)	4.8 (3.7‒6.2)	25.6 (24.0–27.2)	8.7 (7.7‒9.8)	4.1 (3.0‒5.5)
White, non-Hispanic	11.4 (11.0‒11.8)	11.9 (11.1‒12.8)	11.4 (9.5‒13.5)	6.2 (5.6‒6.9)	27.1 (26.3–27.9)	9.2 (8.9‒9.6)	9.1 (7.6‒10.8)
Asian, non-Hispanic	4.3 (3.5‒5.4)	3.9 (2.7‒5.6)	4.5 (2.8‒6.8)	1.9 (1.1‒3.3)	18.5 (15.8–21.4)	1.9 (1.4‒2.5)	1.3 (0.5‒2.8)
**FPL^¶¶^**							
≤100% FPL	11.0 (10.1‒12.1)	9.6 (8.2‒11.2)	11.3 (9.3‒13.6)	6.3 (5.2‒7.7)	26.8 (25.4–28.2)	8.8 (8.0‒9.7)	7.2 (5.6‒9.2)
>100% to ≤200% FPL	9.8 (9.0‒10.7)	9.7 (8.5‒11.0)	11.0 (8.9‒13.5)	5.8 (4.9‒6.8)	25.1 (23.9–26.4)	8.0 (7.3‒8.8)	6.3 (4.9‒8.1)
>200% FPL	9.9 (9.5‒10.3)	9.6 (8.9‒10.3)	9.4 (7.8‒11.3)	4.7 (4.2‒5.3)	25.8 (25.0–26.7)	7.3 (7.0‒7.6)	7.0 (6.6‒10.0)
Highest level of parent education***							
Less than high school	8.7 (7.3‒10.4)	6.5 (5.1‒8.4)	6.2 (4.5‒8.3)	4.6 (3.4‒6.1)	NA	7.7 (6.2‒9.4)	3.4 (1.8‒5.8)
High school graduate	9.8 (9.0‒10.8)	8.1 (6.8‒9.5)	12.5 (9.9‒15.6)	4.4 (3.5‒5.5)	NA	8.6 (7.8‒9.4)	8.2 (5.9‒11.0)
More than high school	10.4 (10.0‒10.7)	10.0 (9.4‒10.8)	10.0 (8.5‒11.7)	5.3 (4.8‒5.9)	NA	7.6 (7.3‒7.8)	7.0 (5.9‒8.3)
**Health insurance^†††^**							
Yes							
Any public	13.1 (12.4‒13.9)	11.4 (10.4‒12.4)	13.0 (11.2‒15.1)	6.6 (5.9‒7.5)	27.6 (26.5–28.6)	10.4 (9.8‒11.1)	8.6 (7.0‒10.4)
Any private	9.4 (9.0‒9.7)	8.5 (7.6‒9.5)	7.7 (6.2‒9.4)	4.5 (3.8‒5.3)	25.5 (24.7–26.3)	7.1 (6.8‒7.5)	5.6 (4.4‒7.1)
No insurance	5.8 (4.7‒7.1)	5.5 (3.8‒8.0)	4.1 (1.9‒7.6)^§§§^	3.0 (2.0‒4.5)	19.8 (17.1–22.9)	4.9 (3.9‒6.3)	0.9 (0.2‒2.4)
**Geographic classification^¶¶¶^**							
Urban/Suburban	9.9 (9.5‒10.4)	9.5 (8.9‒10.2)	NA	5.1 (4.7‒5.6)	26.1 (25.4–26.8)	7.4 (7.1‒7.8)	NA
Rural	10.2 (9.4‒11.1)	10.3 (8.9‒12.0)	NA	6.0 (4.9‒7.3)	24.5 (23.2–25.9)	10.3 (9.4‒11.1)	NA

**Abbreviations**: FPL = federal poverty level; NA = not available; NHANES = National Health and Nutrition Examination Survey; NHIS = National Health Interview Survey; NSCH = National Survey of Children’s Health; NSDUH = National Survey on Drug Use and Health.

* “During the past 12 months, has this child received any treatment or counseling from a mental health professional?“

^†^ *NHIS:* “During the past 12 months, have you seen or talked to...a mental health professional such as a psychiatrist, psychologist, psychiatric nurse, or clinical social worker...about child's health?“ *NHANES:* “During the past 12 months, has the child seen or talked to a mental health professional such as a psychologist, psychiatrist, psychiatric nurse or clinical social worker about their health?“

^§^ “Did you see or talk to this general doctor because of an emotional or behavioral problem that [child] may have?“

^¶^ Receipt of specialty and nonspecialty mental health services.

** “During the past 12 months, has this child taken any medication because of difficulties with his or her emotions, concentration, or behavior?“

^††^ Use of psychotherapeutic agents in past 30 days.

^§§^ Estimates exclude other race and ethnicity groups that did not have a large enough sample size to produce stable estimates.

^¶¶^ For NSCH and NHIS, FPL is based on family income and family size using the Census Bureau’s poverty thresholds for the previous calendar year. Imputed income files were used to impute family income when it was not provided, and for NSCH, family size was imputed using other information about the household when the number of family members was not provided (https://www.census.gov/topics/income-poverty/poverty/guidance/poverty-measures.html). NSDUH only imputes family size when exact counts cannot be determined from the household roster. NHANES uses the US Department of Health and Human Services poverty guidelines in calculating the FPLs (also known as the family income to poverty ratio) and does not impute missing incomes.

*** The highest level of parent education is based on the highest education level among up to two adults who were identified as primary caregivers in the survey. For NHANES, education of household reference person and spouse of household reference person (most often primary caregiver of youth).

^†††^ Private included any insurance from an employer or union, directly purchased, TRICARE or other military health care, or the Affordable Care Act; coverage from any government assistance plan was considered public, including Medicaid or other state-sponsored health plans including the Children’s Health Insurance Program. Respondents who indicated both public and private insurance coverage were represented in both subcategories.

^§§§^ Estimate did not meet all NCHS data presentation standards (CI width >5, relative CI width >130) and should be interpreted with caution.

^¶¶¶^ Method for determining geographic classification differed by survey. *NSCH:* 2010 Office of Management and Budget metropolitan and micropolitan statistical areas standards (https://www.govinfo.gov/content/pkg/FR-2010-06-28/pdf/2010-15605.pdf). Urban/suburban includes metropolitan statistical areas associated with at least one urbanized area of at least 50,000 population; rural was defined as counties that were not part of a metropolitan statistical area; *NHIS:* 2013 NCHS urban/rural classification (https://www.cdc.gov/nchs/data/series/sr_02/sr02_166.pdf). Urban/suburban includes large metropolitan, medium metropolitan, and small metropolitan areas, whereas rural includes nonmetropolitan areas with >50,000 population; *NSDUH:* Rural-Urban Continuum Codes (https://www.ers.usda.gov/data-products/rural-urban-continuum-codes/). Urban/suburban includes large metropolitan, medium metropolitan, and small metropolitan areas; rural includes nonmetropolitan counties.

Regarding medication, 2016–2019 NSCH data indicated that 7.8% of children and adolescents aged 3–17 years had taken medication because of difficulties with emotions, concentration, or behavior during the past 12 months, and 2013–2018 NHANES data indicated that 6.6% of children and adolescents aged 3–17 years had used a psychotropic medication during the past 30 days (Table 12).

State prevalence estimates for mental health treatment from a health professional among children and adolescents aged 3–17 years based on 2016–2019 NSCH data ranged from 6.5% in Nevada to 15.6% in Montana (Supplementary Table 3; https://stacks.cdc.gov/view/cdc/113924). NSDUH 2018–2019 data indicated that receipt of mental health services among adolescents aged 12–17 years ranged from 15.9% in Tennessee to 35.9% in Colorado. NSCH 2016–2019 data indicated that the prevalence of using medications related to mental health in the past year among children and adolescents aged 3–17 years ranged from 3.7% in California and Nevada to 12.4% in Louisiana.

Consultations with general health care providers and mental health professionals about emotional or behavioral problems, as well as use of medications for mental health problems, increased with age. NHIS data from 2017–2018 for children and adolescents aged 3–17 years showed that boys were more likely than girls to have seen a mental health professional during the past 12 months and that parents of boys were more likely to have spoken with a doctor about a child’s emotional or behavioral problem. NSCH 2016–2019 estimates of mental health treatment by a professional among boys aged 3–17 years (10.6%) and girls (9.6%) did not differ. However, NSDUH 2018–2019 data indicated that a higher proportion of adolescent girls aged 12–17 years received mental health services compared with adolescent boys. Data from both NSCH 2016–2019 and NHANES 2013–2018 showed that boys aged 3–17 years were more likely than girls to take medications related to mental health (i.e., for difficulties with emotions, concentration, or behavior or a psychotropic medication).

Across surveys (NSCH, NHIS, NSDUH, and NHANES), for each indicator of mental health services, White children had the highest estimated prevalence, whereas Asian children had the lowest estimated prevalence. NSCH data from 2016–2019 for children and adolescents aged 3–17 years indicated that Hispanic children had a lower estimated prevalence of taking mental health-related medication than White and Black children; a similar pattern was indicated by NHANES 2013–2018 data for children and adolescents aged 3–17 years, although the estimates for Black and Hispanic children did not differ. No consistent patterns were noted for mental health service variables by household poverty level. Children and adolescents aged 3–17 years (NHIS) or 4–17 years (NHANES) of parents with less than a high school education were less likely to have a mental health consultation with a professional than children of parents with more than a high school education; for NHANES, children of parents with a high school education were also less likely to have a mental health consultation with a professional than children of parents with a high school education. NHANES data indicated that children and adolescents aged 3–17 years of parents with less than a high school education were least likely to have used a psychotropic medication during the past 30 days; however, NSCH data did not reflect this difference, and the prevalence of other mental health service variables did not vary by parent education level. The prevalence of each indicator of mental health services was higher for children with public health insurance compared with children with private health insurance, although the prevalence estimates from NHANES for current medication did not differ. All prevalence estimates for mental health services were higher for children with health insurance versus with no health insurance, although the prevalence estimate from NHANES and NHIS for receiving mental health services among children with no health insurance did not differ from children with private health insurance; the estimate for a mental health consultation with a general physician in NHIS data also did not differ for children aged 3–17 years with private health insurance compared with those with no health insurance. NSCH data indicated that use of mental health–related medication among children and adolescents aged 3–17 years was higher among children and adolescents living in rural versus urban areas, whereas prevalence estimates of consulting with a mental health professional or with a general physician about mental health problems were similar among children and adolescents living in rural and urban areas.

### Positive Indicators of Mental Health

Positive indicators of mental health are factors that are associated with good mental health, including emotional well-being, effective regulatory and coping skills, and supportive social relationships (*67*,*68*). Understanding positive indicators of mental health can provide important information about characteristics of children, such as behaviors and skills, that can be promoted to improve overall outcomes, including mental health, physical health, social relationships, and education (*4*–*7*,*67*). For example, positive socioemotional skills such as self-regulation and resilience can be enhanced through intervention and predict long-term positive outcomes (*26*,*106*).

NSCH (2018–2019) assessed six positive indicators of mental health for children aged 6 months–17 years. Parents were asked to report how often their child exhibited certain behaviors, and responses were categorized as “usually or always” versus “sometimes or never.” Among children aged 6 months–5 years, 97.3% usually or always showed affection (i.e., were affectionate and tender), 89.8% usually or always showed resilience (i.e., bounced back quickly when things did not go their way), and 99% usually or always showed positivity (i.e., smiled and laughed a lot) (Table 13). Among children aged 6 months–17 years, 91.3% usually or always showed curiosity (i.e., interest and curiosity in learning new things). Among children aged 6–17 years, 84.5% usually or always showed persistence (i.e., worked to finish tasks after starting), and 76.8% usually or always showed self-control (i.e., stayed calm and in control when faced with a challenge).

Among children aged 6 months–5 years in every state, ≥90% were reported to show affection, ranging from 91.5% in Arizona to 99.4% in Colorado and North Carolina (Supplementary Table 4; https://stacks.cdc.gov/view/cdc/113924). The estimated prevalence of resilience among children aged 6 months–5 years ranged from 84.0% in Arizona to 96.1% in New Hampshire. Among children aged 6 months–5 years in every state, ≥96% were reported to show positivity. The estimated prevalence of curiosity among children aged 6 months–17 years ranged from 87.0% in Oregon to 94.5% in Maryland. Persistence among children aged 6–17 years ranged from 77.2% in Mississippi to 90.5% in New Jersey. Among children aged 6–17 years, the estimated prevalence of self-control ranged from 66.8% in West Virginia to 81.6% in New York.

The prevalence of positive indicators of mental health varied by sociodemographic characteristics. Prevalence of curiosity was higher among children aged 3–5 years and 6–11 years than among children aged 12–17 years, whereas the prevalence of self-control was higher among adolescents aged 12–17 years than among children aged 6–11 years. The percentage of girls who were reported to demonstrate curiosity, persistence, and self-control was higher than the corresponding percentage of boys. No differences by race and ethnicity were found in the prevalence of affection or positivity for children aged 6 months–5 years. White children aged 6 months–5 years had a higher prevalence of resilience than Hispanic, Black, and Asian children; no differences were found between the other groups. White children aged 6 months–17 years had a higher prevalence of curiosity than Hispanic and Black children, and White and Asian children had a higher prevalence of persistence than Black children. Among the four groups studied, the highest prevalence of self-control among children aged 6–17 years was among Asian children.

Children who had a parent with more than a high school education had higher parent-reported ratings on each of the positive mental health indicators than those whose parents had lower levels of education, although no difference was found in rates of affection and positivity among children whose parents had more than a high school education compared with those whose parents had less. Children with private health insurance had a higher estimated prevalence of each positive mental health indicator than children with public health insurance, except for positivity, which did not differ by health insurance status. For each of the other five indicators, children with no health insurance had prevalence estimates between or similar to those of children with private and public health insurance. A linear pattern was noted for poverty level; children living in households with the highest income level (>200% FPL) were most likely to demonstrate each indicator, except for positivity. Among the groups studied, the lowest prevalence of curiosity, persistence, and self-control was among children living in households with the lowest income level (≤100% FPL). Only one indicator varied by geographic classification; children living in urban areas had a higher estimated prevalence of self-control than those in rural areas.

## Discussion

Surveillance of children’s mental health and mental disorders is a critical part of defining the overall public health impact, increasing awareness about mental health and mental disorders, and identifying potential needs for allocation of resources. Overall, no single, comprehensive surveillance system for children’s mental health in the United States exists, and no single indicator can be used either to define what constitutes mental health in children or to identify the number of children with a mental disorder. These data confirm the overall finding from the previous report (*8*), which is that mental disorders among children continue to be a substantial public health concern.

One finding from the surveillance systems that collect data on children’s mental health was that mental disorders can begin in early childhood. Approximately 2% of children aged 3–5 years had ever received a diagnosis of ADHD, anxiety, or ASD, according to parent-reported data from NHIS 2017–2018 and NSCH 2016–2019, and approximately 5% had ever received a diagnosis of behavior problems. In contrast, NSCH data indicated that most young children were reported to show affection and positivity. Across data sources, estimates of mental disorders (i.e., ADHD, depression, anxiety, ASD, and Tourette syndrome) and receipt of mental health services generally increased with age, although reports of behavior problems decreased among adolescents aged 12–17 years. Suicide (2018–2019 NVSS and 2014–2018 NVDRS) was more common among older adolescents aged 15–19 years than children and younger adolescents aged 10–14 years. Although the positive indicator of curiosity decreased with age, the prevalence of persistence was similar among children in the age groups of 6–11 years and 12–17 years, and the prevalence of maintaining self-control was higher among adolescents aged 12–17 than among children aged 6–11 years. These data show the advantage of surveillance systems that include mental health indicators for children of all ages because of their ability to detect differences by age.

Mental disorders affect children across the range of sociodemographic characteristics; however, the prevalence varies by certain characteristics. Consistent with previous studies, boys had a higher prevalence than girls of ADHD, behavioral or conduct problems, ASD, Tourette syndrome, and rates of suicide (*1*,*34*,*38*,*42*,*82*,*86*), and girls had higher estimated prevalences of depression, suicidal ideation, and attempted suicide (*1*,*34*). Girls also had a higher estimated prevalence of curiosity, persistence, and self-control than boys. Although previous studies have shown higher a prevalence of anxiety among females compared with males (*1*,*34*), previous estimates with NSCH data have shown no differences in anxiety by sex (*17*,*42*), similar to the results in this report. Although the reported prevalence of substance use and substance use disorders have tended to be higher in boys than girls (*1*,*34*), no differences by sex based on 2018–2019 NSDUH data were found for substance use disorder or illicit drug use disorder, and girls had a slightly higher estimated prevalence of past year alcohol use disorders than boys. These findings are similar to findings based on 2010 NSDUH data (*107*). Other studies have shown that patterns of drug use by sex also differ by age group and type of drugs used as well as changing trends in the use of specific drugs (*29*,*40*,*108*). Data from the 2016–2019 NSCH and 2017–2018 NHIS indicated that boys were more likely than girls to have had a mental health consultation or received medication for mental health problems, whereas NSDUH data indicated that more girls than boys reported receiving mental health services.

The prevalence of many mental disorders and indicators differed by race and ethnicity. Black and White children had the highest prevalence of ADHD, and Black children also had the highest prevalence of behavioral or conduct problems. Because evidence has shown that racial bias can result in certain behaviors among Black children being incorrectly interpreted as disruptive, this finding might represent overdiagnoses or misdiagnoses that are masking other forms of mental distress among Black children (*21*,*22*). The findings that Black and White children had the highest prevalence of ADHD and that the estimates for these two groups were similar are different from findings from older studies, which found that White children had a higher prevalence of ADHD than children from other racial and ethnic groups. Recent studies have reported a higher prevalence of ADHD and of ADHD combined with a learning disability among Black children than among White children, suggesting that this association might be changing (*36*,*38*). Similarly, previous studies based on NSCH data have reported a higher prevalence of Tourette syndrome among White children than among Hispanic and Black children (*109*), whereas 2016–2019 NSCH data in this report indicated a similar estimated prevalence of Tourette syndrome among White and Hispanic children. Although future years of data can be used to confirm whether these differences between White and Black children have persisted, these patterns, combined with the data on ADHD, suggest that diagnoses of these disorders might have increased among Black and Hispanic children in recent years, a finding that has also been noted for developmental disabilities (*110*).

White children had the highest estimated prevalence of anxiety, each indicator of mental health services use, and the positive indicator of resilience. YRBS 2019 data indicated that Hispanic and AI/AN high school students primarily aged 14–18 years had the highest prevalence of feeling sad and hopeless, although the NSCH 2016–2019 data indicated that the prevalence of diagnosed depression among Hispanic children and adolescents aged 3–17 years was similar to or lower than the prevalence among White children. Data from 2018–2019 NSDUH indicated a similar prevalence of MDE among Hispanic and White adolescents aged 12–17 years, with both groups having a higher prevalence of MDE than Black adolescents. Similarly, the NSDUH prevalence estimate of alcohol use disorder was higher among White adolescents than among Black and Asian adolescents; no significant differences by race and ethnicity were found for prevalence of illicit drug use disorder. Other studies have found that for numerous years, Black adolescents have reported substantially lower levels of illicit drug use than White and Hispanic adolescents; however, the difference has decreased in recent years (*111*).

Black adolescents aged 12–17 years had a lower prevalence of MDE than White adolescents based on NSDUH 2018–2019 data, whereas for other indicators of depression, including diagnosed depression among children and adolescents aged 3–17 years using 2016–2019 NSCH data, the prevalence was similar for Black and White children. Also based on NSCH data, Black children tended to have a lower prevalence of anxiety problems than White children, and the prevalence was more similar between Black and Hispanic children. Although the prevalence of receipt of mental health services was consistently lower across data sources (2016–2019 NSCH, 2017–2018 NHIS, 2018–2019 NSDUH, and 2013–2018 NHANES) for Black children compared with White children, the prevalence was only different for three of the mental health service indicators: mental health treatment by a professional (NSCH data), mental health consultation with a professional (NHIS data), and current medication for mental health problems (NHANES data). Black children and children in other minority groups are at increased risk for poor mental health related to the known impact of structural and individual racism on mental health and access to services (*20*,*112*).

Because a substantial subset of Hispanic children in the United States are first- or second-generation immigrants (*113*), increased levels of depression in the Hispanic population might be related to migration stressors for first-generation youths (*114*), parental citizenship status (*115*), and cultural differences (*116*). However, they also might be associated with experiences related to racism and bias against cultural minorities, such as bullying experiences (*117*,*118*). At the same time, Hispanic children might be less likely than non-Hispanic children to access mental health care services, which might result in lower rates of diagnosis (*119*). Estimates of ASD by race and ethnicity varied by data source and time frame (e.g., current ASD versus ever receiving an ASD diagnosis). Although Hispanic children had a higher prevalence of ever having received an ASD diagnosis and currently having ASD than Asian children based on 2016–2019 NSCH data, the estimate of ASD was higher for Asian children than Hispanic children based on 2016 ADDM Network data. Asian children had the lowest prevalence of ADHD, behavioral or conduct problems, depression (NSCH), anxiety, ASD (except for ADDM Network data, in which Hispanic children had the lowest prevalence), and receipt of mental health services and the highest prevalence of self-control. Previous studies have shown increased risk for poor mental health among Asian children (*120*,*121*), although findings have been mixed (*122*,*123*). Research on Asian children is limited and might be complicated by other factors, including heterogeneity on factors such as cultural group, immigration status, socioeconomic status, or methodological factors such as sampling, sample size, and recruitment (*47*,*120*,*121*). Data are even more limited for AI/AN and NH/OPI children; only data on suicide (from 2019 YRBS, 2018–2019 NVSS, and 2014–2018 NVDRS) and depression (from 2018–2019 NSDUH and 2019 YRBS) were included because the sample sizes for AI/ANs and NH/OPIs were too limited to generate estimates from the other data systems. AI/AN persons have increased risk factors for poor mental health, a higher prevalence of mental disorders, and limited access to behavioral health care (*124*–*126*); therefore, surveillance that includes a sufficient number of AI/AN children would allow for better monitoring of the mental health of this population (*124*). Additional data and analyses are needed to better understand differences between and within racial and ethnic groups, as well as which specific factors contribute such differences. 

The findings in this report show an association between health disparities and a lack of economic resources with indicators of children’s mental health (*2*). Children living in households with the lowest income level (≤100% FPL) had the highest prevalence of ADHD, behavior or conduct problems, depression (NSCH and NHANES, lowest for NSDUH), and anxiety and tended to have the highest prevalence of use of mental health services (with the exception of the NHIS question about seeing a mental health professional, which did not vary by poverty level). Conversely, children living in households with the highest income level (>200% FPL) tended to have a higher prevalence of the various positive mental health indicators and a lower prevalence of ASD. The prevalence of ADHD and behavior problems was lower among children with parents who had more than a high school education than among those whose parents’ highest level of education was high school, whereas anxiety and positive indicators of mental health were highest among children of parents with more than high school education. Children with public health insurance had a higher prevalence of ADHD, behavior problems, diagnosed depression, anxiety, ASD, and receipt of mental health services and a lower prevalence of five of the six positive mental health indicators (all except positivity).

Previous studies of the impact of geographic classification indicated that children living in rural areas have increased risk factors for poor mental health and barriers to accessing mental health services (*37*,*127*). For example, children living in rural areas are more likely than children living in urban areas to have poor physical health, exhibit health risk behaviors, and live in communities with more adults who have poor mental health (*37*,*128*,*129*). Data in this report show that children living in rural areas were more likely to have a diagnosis of ADHD, behavior problems, depression, and anxiety and were more likely to have used mental health–related medication than children living in urban areas. No differences in use of other mental health services were found between children in urban versus rural areas. These findings are similar to those from previous studies showing that children in rural areas are more likely to use medication but might be less likely to receive behavioral treatment (*127*), which might be related to a shortage of mental health providers in rural areas (*130*).

### Limitations

The findings in this report are subject to at least eight limitations. First, numerous gaps exist in surveillance of mental disorders and mental health among U.S. children, many of which were described in the 2013 report (*8*). Existing surveillance covers a limited number of disorders and does not allow for estimates of many specific disorders that occur among children, including obsessive-compulsive disorder, posttraumatic stress disorder, or bipolar disorder. Furthermore, the majority of surveillance systems rely on parent report of a previous diagnosis, which is influenced by access to health care to receive a diagnosis, the parent’s ability to recall the diagnosis and willingness to report the diagnosis, and the accuracy of the diagnosis itself. Health care providers might differ in how they apply diagnostic criteria and distinguish among different diagnoses. Although anxiety is one of the most common mental disorders in children (*34*,*131*), only NSCH provides regular data on the prevalence of anxiety in U.S. children. Relying on parent reports of diagnoses can be particularly challenging with emotional disorders such as anxiety that might be better assessed by self-report, particularly among adolescents (*132*,*133*). Lack of data on anxiety in other surveys might limit understanding of this disorder in relation to other indicators such as suicidality and other youth risk behaviors.

Second, data from these surveillance systems cannot be used to provide overall estimates of good or poor mental health. Data on positive indicators of mental health, which were only assessed in NSCH, are limited. Furthermore, of the positive indicators that were included, three were only available for children aged ≤5 years, two were available for children aged 6–17 years, and only one indicator (curiosity) was available for children aged 6 months–17 years. Although these items were developed to describe positive development, data are limited on whether these capture the intended constructs with sufficient breadth and depth (*67*,*68*). In addition, whether the items favor positive attributes among White children is unknown, nor is whether these positive characteristics are more likely to be inherently identified among persons from higher socioeconomic groups. Specific factors related to positive indicators, such as child attachment to the parent (*68*), might require more in-depth assessment (*134*). Finally, the only negative indicator of a mental health crisis that is routinely captured is suicidality; data on other forms of crises, such as need of emergency care for mental health, are important indicators but are not collected in population-based surveillance systems (*135*). This limits the ability to understand the full spectrum of mental health and of both diagnosed and undiagnosed mental disorders.

Third, even when indicators of mental health and mental disorders are available, various factors limit the use of these data to understand differences in certain populations or to understand the complete context of mental health and mental disorders, including possible health disparities based on social determinants of health. In many cases, sample sizes are inadequate for description by racial and ethnic categories, by state, or by other specific indicators. For example, even with 4 years of NSCH data combined, estimates for Tourette syndrome among Black and Asian children and among children with parents who have lower education levels or children with no health insurance were not reliable and could not be included in this report. Furthermore, despite using larger data sets, only four racial and ethnic groups were consistently reported among the different surveys, limiting understanding of differences in mental health and mental disorders among other racial and ethnic groups or among subgroups of racial and ethnic groups, such as comparing persons in minority groups who have recently immigrated with those whose ancestors have lived in the United States for generations. In addition to differences in prevalence by sociodemographic characteristics, previous research has shown that mental disorders and poor mental health are more common in certain groups of children, including those with other disabilities and chronic health conditions (*136*), children affected by racism (*20*,*137*) and other adverse childhood experiences (*138*), and lesbian, gay, bisexual, and transgender (LGBT) children (*139*). NSCH collects information on disabilities, chronic conditions, and some adverse childhood experiences, including perceived racial discrimination, unstable relationships, and exposure to violence (*140*); thus, these associations can be explored in the future. Although some data systems (e.g., YRBS) collect information about LGBT status, too few systems collect these data to be included in this report. Collection of more data on health equity indicators, such as structural and individual racism, sexual orientation and gender identity, adverse experiences, and other sociodemographic characteristics related to social determinants of health (*23*), might highlight additional opportunities to promote health equity (*24*).

Fourth, surveillance estimates for mental health at the population level are not timely enough to monitor immediate changes in mental health of a population before, during, and after public health emergencies, such as hurricanes or the COVID-19 pandemic (*141*). In response to COVID-19, the Household Pulse Survey was implemented quickly and included questions on adult mental health that were comparable to the NHIS questions (*142*). Other surveys were developed that included questions on adult mental health (*143*,*144*), whereas fewer considered children’s mental health (*145*,*146*), and they lacked baseline data from before the COVID-19 pandemic for comparison. In an attempt to document changes in mental health related to the pandemic, the Coronavirus Health and Impact Survey asked participants, including parents and children (via self-report for those aged 9–18 years), to report on their mental health status before the pandemic (*147*). Data from the National Syndromic Surveillance Program allowed for near real-time estimates of mental health–related emergency department visits for both children and adults and comparisons with estimates before the COVID-19 pandemic. However, these data only include problems that resulted in a visit to an emergency department and therefore do not represent the mental health status of the entire population of children (*135*,*148*). In public health emergencies, immediate actions are needed to protect children’s mental health; thus ongoing, timely surveillance might provide benchmark data for comparison with data collected during and after an event to guide response actions and resource management.

Fifth, each surveillance system is designed for specific purposes and uses different methods, affecting the ability to compare estimates directly across systems or integrate findings across systems. For example, NSCH includes questions about diagnosed anxiety and depression but not specific questions about treatment for anxiety and depression. NHANES has data on use of specific psychotropic medications but not on diagnosis of anxiety or depression, which means that these factors cannot be compared. Different systems focus on different ages, use different methods for sampling and data collection, and include different indicators of mental health and mental disorders. In addition, other indicators vary across systems, including how geographic classification is defined, the availability of data for different racial and ethnic groups, and year of data collection. These differences limit the ability to determine how much of the variability in estimates is due to differences in survey methods, sociodemographic characteristics, and other unmeasured factors (e.g., clinical information and cultural context).

Sixth, the data collected in these systems are cross-sectional, focusing on point estimates of mental health and mental disorders, and only allow for estimations of association, not causation. Much of the data are based on parent or adolescent self-report, including report of health care provider diagnosis, and are subject to recall error and inaccurate or incomplete diagnoses and disparities in access to health care. Moreover, parent-reported data might be influenced by parent perspective and other factors, including parent mental health. Cultural differences might also affect parent reporting; parents might differ in how they interpret or report on items such as whether a child currently has a condition or behavior. For example, determining whether racial and ethnic differences in child resilience, curiosity, and persistence are influenced by cultural differences in how the behaviors are interpreted is not possible. Stigma related to mental health might also influence parent and adolescent reporting and the likelihood of seeking care, a concern consistent with emerging evidence of potential negative effects of receiving an ADHD diagnosis on some children (*149*,*150*). The data are subject to errors such as sampling and nonsampling errors, including measurement errors or nonresponse bias, although survey estimates are weighted for nonresponse.

Seventh, the data presented in this report also are limited in scope and focus; data are collected at a single time point and do not represent information on trends in prevalence over time. Previous reports have used data from these systems to document increases in ADHD, ASD, anxiety, MDE, and suicidal behaviors (*17*,*38*–*40*,*85*,*151*). Monitoring changes in prevalence over time is critical to understanding the public health impact related to mental health and mental disorders. All data included in this report were collected before the COVID-19 pandemic and do not reflect the impact of the pandemic on children’s mental health.

Finally, the data are presented to show how specific sociodemographic characteristics are associated with specific mental disorders; however, the complexity of the relation between the characteristics and disorders is not adequately described. Specifically, disorders might vary by sex and age together, such that estimates of anxiety and depression are typically similar by sex in younger children but in adolescents are more common in girls than boys (*17*,*152*,*153*). In addition, the presented data do not describe whether children had more than one disorder, which is common and has implications for health care needs and health outcomes (*1*,*34*,*75*). Socioeconomic indicators are likely to be highly correlated. For example, because public health insurance is linked to poverty, the protective effect of public health insurance for families with low economic resources might not be apparent unless income is considered. Moreover, because of the significant association of poverty and racial and ethnic minority status in the United States, additional research could examine the extent to which differences in rates of indicators experienced by different racial and ethnic groups persist when access to resources is considered or whether they are better explained by health disparities based on racial bias, by cultural differences, or by other factors. Similarly, additional research might help clarify the extent to which rural status represents structural inequities related to disparities in access to services and support or to lack of financial resources or is confounded by racial and ethnic differences and disparities (*112*,*154*). These complex interactions are beyond the scope of this report, which provides a broad perspective on surveillance systems that provide information on children’s mental health.

### Current Efforts to Improve Surveillance of Children’s Mental Health

Despite the identified challenges in surveillance related to children’s mental health, numerous efforts have been implemented and are underway to improve the availability of related data. As noted previously, the design and administration of NSCH has changed substantially since the 2013 report on this topic. The goals and process of the redesign have been described elsewhere (*66*). Decreasing response rates, an increasing proportion of households without a landline telephone, and the desire to provide more timely data prompted HRSA MCHB to transition NSCH from an interviewer-assisted telephone survey conducted quadrennially to a self-administered annual survey using online and paper-based methods. Attempts were made to retain all content necessary to produce important estimates related to child health and well-being, including the survey items used for analyses presented in this report. However, whereas the questions remained the same, based on the results from mode effects and operational tests, HRSA MCHB has determined that making direct comparisons to data collected before 2016 is not possible.

HRSA MCHB is committed to the annual assessment of the NSCH indicators presented in this report. The annual administration of NSCH presents opportunities to continue testing the framing and wording of these existing items through regular cognitive and usability testing, as well as the assessment of new content on emergent and persistent mental, emotional, and behavioral problems among children and adolescents (e.g., disordered eating). After 2016, NSCH added items to more specifically address positive child development in the preschool years using the Healthy and Ready to Learn indicators (*155*). These indicators include two composites that are closely associated with mental health, self-regulation and social-emotional development, which might provide ways to identify groups of children who need additional support (*155*,*156*). Finally, HRSA MCHB is exploring options to further investigate the specific challenges presented as a result of the COVID-19 public health crisis through NSCH.

NHIS was redesigned in 2019 to reduce the time required of respondents and to make the content more relevant to the data needs of the U.S. Department of Health and Human Services. To reach these goals, the survey length was reduced by implementing rotating content with fixed periodicity alongside annual content. Rotating content on a fixed periodicity schedule allows for more content to be included over the long term; for data users, partners, and communities to be able to plan for data availability; and for a shorter interview for the respondent. A panel of technical experts in the field of children’s health convened, consisting of stakeholders representing federal government as well as academic and public health researchers, and identified the enhancement of content related to children’s mental health as a priority for the redesigned NHIS. As such, mental health assessment, specifically the full Strengths and Difficulties Questionnaire and impact questions, and mental health care use, which includes use of medication, receipt of services, and reduced access to mental health care due to cost, will be measured on a rotating basis. In addition, beginning in 2019, selected stressful life events and expanded social determinants of health were added to the survey, which are useful measures to assess in conjunction with mental health measures. The Baby Pediatric Symptom Checklist, a 12-item validated screening tool, was added to NHIS annual core to assess social and emotional difficulties among children aged 0–23 months (*157*). Also, in 2019, the Washington Group/UNICEF Module on Child Functioning (*158*–*160*) was added to NHIS to measure functional limitations in children aged 2–17 years annually; NHANES has included some of the questions for children aged 5–17 years since 2019. (Data for the 2019–2020 cycle were insufficient to generate nationally representative estimates and will only be available for analysis without sample weights. Therefore, nationally representative data might not be available for these indicators until the 2021–2022 data have been collected and released.) The questions ask about a range of functional limitations, including the child’s level of difficulty concentrating, ability to accept changes in routine, and ability to make friends, as well as the frequency of experiencing anxiety and depression among children aged 5–17 years. The new NHIS design allows for the inclusion of emerging public health topics. More information on the periodicity of NHIS content is available online (https://www.cdc.gov/nchs/data/nhis/Sample-Child-Questionnaire-Summary-508.pdf).

Future reports from the ADDM Network will use revised methods to produce more timely and efficient reporting of ASD prevalence, progress toward early identification, and the transition of adolescents with ASD to adulthood. ADDM will continue to incorporate chart review; however, ASD ascertainment will reflect ASD diagnoses or classifications from various medical, educational, and service providers in each participating community. An analysis comparing the previous and new case definitions showed the two produced similar ASD prevalence estimates, and other indicators were essentially unchanged (*161*). The changes enabled ADDM sites to incorporate a wider range of data sources (such as state Medicaid programs), allowed for expanded tracking of early ASD identification among children aged 4 years, and allowed sites to begin reporting on the medical issues, mental health issues, and challenges with the transition to adulthood among children aged 16 years with ASD. Several ADDM sites are also piloting an approach to provide rapid statewide reporting of ASD prevalence, which could provide basic indicators of disparities in services or regional variations in ASD identification practices.

Additional indicators of mental health that address the continuum of good to poor mental health are also being added to surveys. Some states and local education agencies have added questions to their YRBS that address protective factors. For example, some YRBS questionnaires include a question about who respondents would talk to if they felt sad, empty, hopeless, angry, or anxious; a question about whether they feel safe and secure in their neighborhood; and a question about whether they feel connected to their school. NSDUH includes questions that ask adolescents whether they have persons they can turn to when they have a serious problem. Improving overall well-being, in addition to improving health, has been identified as an overarching objective for Healthy People 2030, the goal for the nation’s health in the United States (*46*).

In addition to the national surveillance efforts described, other activities have been conducted to understand the public health impact of children’s mental health and mental disorders since the previous report. The Project to Learn About Youth Mental Health (PLAY-MH), a community-based epidemiologic study, examined symptoms of mental disorders in school-aged children and adolescents in four different U.S. school districts during 2014–2018 (*131*). On the basis of data collected from teachers and parents on children’s symptoms and impairments, approximately one in six students met DSM-IV criteria for having a childhood mental disorder, including anxiety disorders (7.9%–11.2% across sites), oppositional defiant disorder (5.7%–17.3%), and ADHD (5.1%–9.4%) (*131*). The PLAY-MH study offers complementary data on both diagnosed and undiagnosed mental disorders among school-aged children (i.e., kindergarten through 12th grade) in the four participating communities. 

To help address the problems inherent in relying on national-level data to guide state and local decision-making, CDC is examining the constraints and value of applying small-area data estimation methods to the NSCH items on mental disorders. Such methods have been applied to the Behavioral Risk Factor Surveillance System (i.e., CDC’s PLACES project; https://www.cdc.gov/places) to provide local estimates for adult data, and to other content areas in NSCH (*162*). In addition, HRSA MCHB and the Census Bureau offer the opportunity for states to sponsor oversampling of specific populations at the statewide or substate levels to support more targeted assessment, program planning and evaluation to aid in state and local decision-making (*163*). YRBSS allows for state, tribal, territorial, and local school district data. Although CDC conducts a national YRBS (with data presented in this report), CDC provides these sites with technical assistance on surveys and quality assurance oversight allowing for site-specific YRBS data for use by education and health agencies (*164*). These combined efforts could provide more precise local estimates of children’s mental disorders and increase the value of the data obtained via investments in NSCH.

CDC efforts are focused on the longer-term goal of building state and local capacity to collect, analyze, and use surveillance data on children’s mental health to monitor population health and connect more families with effective mental health services. Through two cooperative agreements, CDC is developing tools to assist state and local stakeholders in their efforts to support children’s mental health. A CDC-supported resource released in May 2020 by the National Network of Public Health Institutes (Data Governance for Children’s Mental Health Surveillance: What Is It and Why Does it Matter?) detailed the specific data governance challenges in working with children’s mental health data (*165*). In July 2020, the Public Health Informatics Institute of the Task Force for Global Health released a CDC-supported summary of laws related to child mental health data collection, sharing, and use (*166*). In October 2021, the Public Health Informatics Institute released a playbook to guide the planning and implementation of a child and adolescent mental health surveillance program using existing data sources (*167*). Tools continue to be developed to assist state and local systems to collect and use data to improve children’s mental health.

## Future Directions and Public Health Implications

The findings in this report can be used by public health professionals, health care providers, state health officials, policymakers, and educators to understand the prevalence of specific mental disorders and other indicators of mental health and the challenges related to mental health surveillance. Because of the substantial impact of children’s mental health on the development, overall health, and well-being of children (*4*,*10*–*12*,*15*,*16*), surveillance data to monitor children’s mental health, such as data on differences by sociodemographic characteristics and disability status, issues related to health equity, changes over time, and the relation to public health emergencies, are critical for directing resources to support children and families, including by identifying mental health workforce provider shortages. Current data sources provide information on both diagnosis and treatment but do not indicate whether children and families are receiving adequate or evidence-based treatment. Improved surveillance can be used to move beyond point-in-time prevalence to identify how well children are doing and when they need support and to cover the entire continuum of children’s mental health and functioning from birth through adolescence. Improved surveillance would also go beyond focusing on diagnoses and would include measures of healthy development and resilience as well as symptoms of mental disorders (*67*). In addition to providing information at the national level, more timely surveillance of children’s mental health would provide data that are actionable at state and local levels. A comprehensive public health approach to surveillance would assess policies and programs that support mental health before a diagnosis is needed (*2*). More comprehensive surveillance could help identify which mental health services, including treatment and prevention efforts, are needed and would be most effective for children with varying disorders, risk and protective factors, and circumstances so that public health interventions can be tailored to promote all children’s mental health. Identifying and addressing structural and systemic factors are critical to promoting health equity.

## Conclusion 

Mental health and well-being are important indicators for public health and have been identified as an important component of improving the nation’s health overall and health equity (*46*,*168*). Although current data sources and measures provide some information on specific disorders and indicators of mental health, the data are not sufficient to provide a comprehensive description of children’s mental health in the United States (*46*). However, the surveillance systems reviewed in this report provide useful and actionable information on the status of several children’s mental health indicators. In addition, this report highlights the importance of ongoing surveillance of children’s mental health to help identify which resources are needed to support children and to evaluate progress associated with efforts to improve children’s mental health.

## Footnotes

* *NHIS and NVSS:* 2013 NCHS urban/rural classification (https://www.cdc.gov/nchs/data/series/sr_02/sr02_166.pdf); urban/suburban includes large, medium, and small metropolitan areas, whereas rural includes nonmetropolitan areas with population <50,000. *NSCH:* 2010 Office of Management and Budget MSA and micropolitan statistical area standards (https://www.govinfo.gov/content/pkg/FR-2010-06-28/pdf/2010-15605.pdf); urban/suburban includes MSAs associated with at least one urbanized area of at least 50,000 population; rural is defined as non-MSA. *NSDUH:* Rural-Urban Continuum Codes (RUCC) (https://www.ers.usda.gov/data-products/rural-urban-continuum-codes); urban/suburban includes large, medium, and small metropolitan areas; rural includes nonmetropolitan counties. *YRBS:* MDR (formerly Market Data Retrieval) propriety information, determined by an MDR formula based on the National Center for Education Statistics Locale Code classification and zip code. The urban category includes the urban, suburban, and town groups, and the rural category includes the rural/nonmetropolitan group.
